# The impact of early special educational needs provision on later hospital admissions, school absence and education attainment: A target trial emulation study of children with isolated cleft lip and/or palate

**DOI:** 10.1371/journal.pone.0327720

**Published:** 2025-07-16

**Authors:** Vincent Nguyen, Kate Lewis, Ruth Gilbert, Bianca De Stavola, Lorraine Dearden

**Affiliations:** 1 University College London Great Ormond Street Institute of Child Health, London, United Kingdom; 2 University College London Institute of Health Informatics, London, United Kingdom; 3 University College London Institute of Epidemiology and Health Care, London, United Kingdom; 4 University College London Social Research Institute, London, United Kingdom; Universiti Malaya Fakulti Perubatan: University of Malaya Faculty of Medicine, MALAYSIA

## Abstract

**Background:**

Special educational needs (SEN) provision is designed to help pupils with additional educational, behavioural or health needs. Our aim was to assess the impact of early SEN provision on health and educational outcomes for a well-defined population, pupils with cleft lip and/or cleft palate (CLP) without additional anomalies.

**Methods:**

We used the ECHILD database, which links educational and health records across England. Our target population consisted of children with a recorded diagnosis of CLP without other major congenital anomalies in hospital admission records in ECHILD. We applied a trial emulation framework to define eligibility into our study and investigate the causal impact of SEN provision in the first year of compulsory school (Year 1 – age five/six years) on various health and educational outcomes accumulated by the end of primary education (Year 6 – age ten/eleven years). SEN provision was categorised as: None, SEN Support, and Education and Health Care Plan (EHCP). The outcomes were: unplanned hospital utilisation, medical and unauthorised school absences, persistent absences, and standardised key stage 1 (KS1) and key stage 2 (KS2) mathematics attainment scores. To account for confounding factors affecting the observed associations and estimate the causal effects of early SEN provision on these outcomes, we used three estimating approaches: propensity score-based methods (inverse probability weighting, [IPW]), g-computation, and augmented IPW (AIPW). Causal effects were measured in terms of average treatment effects (ATE) and average treatment effects on the treated (ATT), expressed as rate ratios (RaR) for hospitalisations and absences, risk ratios (RiR) for persistent absences, and mean differences (Δ) for academic scores. Missing values of the confounders were handled via the missing covariate indicator method. We triangulated these results with those obtained by univariable and multivariable regression.

**Results:**

Our study included 6,601 children with CLP and without additional major congenital anomalies. Evaluations involving EHCP were limited by the low numbers of comparative children. Thus, only comparisons of SEN Support (N = 2,009, 31.6%) versus None (N = 4,350, 68.4%) are reported. Observed rates of unplanned hospitalisation (RaR_crude_ = 1.31, 95% confidence interval (CI): 1.12, 1.52), persistent absence (RiR_crude_ = 2.21 (1.87, 2.62)) and medical absence (RaR_crude_ = 1.34 (1.28, 1.40)) were higher amongst children with recorded SEN support, whilst KS1 and KS2 maths scores were lower (Δ _crude_ = −0.85 (−0.90, −0.79) and Δ _crude_ = −0.82 (−0.89, −0.75), respectively). Contrary to the observed relative rates and risks, we found small or no evidence of a causal effect of SEN Support on unplanned hospitalisation (ATE: RaR_IPW_ = 1.16 (1.00, 1.34), RaR_g_ = 0.99 (0.87, 1.12); RaR_IAPW_ = 1.02 (0.87, 1.17) or persistent absences (ATE: RiR_IPW_ = 1.13 (0.92, 1.34); RiR_g_ = 1.08 (0.86, 1.31); RiR_AIPW_ = 1.20 (0.96, 1.45)). We found that SEN support increased rates of medical absences (ATE: RaR_IPW_ = 1.10 (1.04, 1.18); RaR_g_ = 1.09 (1.03, 1.15); RaR_AIPW_ = 1.04 (0.95, 1.13)), decreased those of unauthorised absences (RaR_IPW_ = 0.86 (0.76, 0.97); RaR_g_ = 0.98 (0.86, 1.09); RaR_AIPW_ = 0.80 (0.66, 0.95)) and decreased – but not as extensively as the crude differences suggested- KS1 (ATE: Δ _IPW_ = −0.18 (−0.25, −0.10); Δ _g_ = −0.21 (−0.26, −0.16); Δ _AIPW_ = −0.25 (−0.32, −0.17)) and KS2 maths scores (ATE: Δ _IPW_ = −0.24 (−0.33, −0.15); Δ _g_ = −0.27 (−0.33, −0.21); Δ _AIPW_ = −0.24 (−0.32, −0.17)). Results for the ATT for each of these outcomes were similar to those for the ATE, indicating no observable evidence of heterogeneity of effects by treatment received. Sensitivity analyses confirmed the robustness of these results.

**Discussion:**

In the population of children with CLP without further major congenital anomalies, assignment to receive or not receiving early SEN Support appears to have no harmful impact on the rates of unplanned hospitalisation or persistent absences, but to increase rates of medical absences, whilst reducing rates of unauthorised absences. For the sub-populations of children with key stage results, such hypothetical intervention does not appear to completely reduce the observed disadvantage in KS1 and KS2 mathematics scores. These results relate to the impact of the intention to intervene not the actual delivery of actual SEN Support provision as this information is not available in school administrative records. Furthermore, we cannot discount the impact of unaccounted confounding factors, such as parental education and early home learning environments, particularly for the education attainment results.

## Introduction

### Background to special educational needs

Special educational needs (SEN) provision, as delivered in an educational environment, offers reasonable adjustments for children and young people who need additional health, academic, or behavioural support. This includes children with complex health requirements or learning difficulties. SEN provision may improve both communication skills and behaviour, consequently impacting on children’s ability to participate in educational and social activities in school.

### Evidence gap

Currently, there is limited research on the impact of SEN provision on academic and healthcare outcomes in populations who have a need for SEN provision, which ideally would require randomised controlled trials (RCT). However, because SEN provision is universally available in primary education in England (from age five to eleven years), implementing an RCT design would be unfeasible and possibly unethical for certain groups of children. In lieu of RCTs, observational studies based on administrative databases provide a pragmatic alternative, but these require careful study design to minimise the risk of bias. A major challenge is the risk of confounding, in particular confounding by indication, whereby assignment to treatment (or, in this case, SEN provision) is systematically related to factors, such as the severity of the condition or the need for intervention [1], that are also likely to be related to health and educational outcomes of interest. Therefore, comparing all children who receive SEN provision against a control group of those who do not have provision would likely lead to erroneous results as the groups are not exchangeable or comparable in terms of the need for provision. Thus, comparisons should be made in homogenous subgroups which are exchangeable in terms of the need for SEN provision with clearly defined outcomes. Other biases may arise from using administrative data to design observational studies because of inappropriate handling of the source databases, for example selection bias if the eligibility criteria are not targeting the population of interest, and immortal time bias if, for example, the timing of eligibility assessment, exposure status, start of follow-up and treatment designation are not aligned. The introduction of such biases can potentially be avoided by adopting the target trial emulation framework [2]. This consists of first designing an ideal (pragmatic) RCT to address the question of interest, and then emulating it by adapting its protocol to the available observational data (Hernán and Robins, 2016).

### Overview of cleft lip and/or palate epidemiology and why we focus on it

In this study we focus on children with cleft lip and/or palate (CLP), a craniofacial birth defect that occurs when the upper lip (cleft lip) or palate (cleft palate) or both failed to join during pregnancy. On average, CLP anomalies are identified in 15 in 10,000 new-borns in England yearly. CLP affects communication (hearing and speech), dental health [3] and psychosocial health and are associated with reduced academic attainment [4] and with increase in hospitalisations when compared to those without CLP [5]. Previous observational evidence of children with CLP in England reported reduced attainment across primary education at higher provision of SEN compared to peers without CLP; they also hypothesised that extra support at the beginning of compulsory education may benefit educational attainment of children with CLP [4]. However, there is limited to no literature assessing the impact of SEN provision on unplanned hospital utilisation and school absences.

### Study aims

In this study, we describe how we used the ECHILD dataset to design a study that emulates an RCT of children who were born with cleft lip and/or palate (CLP) with the aim of addressing the causal question of the impact of receiving alternative categories of SEN provision (including none) on a number of health and educational outcomes accumulated by the end of primary education (Year 6 – age ten/eleven year). These outcomes are unplanned hospital utilisation, medical and unauthorised school absences. In addition, we examine the impact of SEN provision on key stage 1 (KS1) and key stage 2 (KS2) results for the children who were followed up to the times of these assessments.

## Methods

### Ethics and consent

The data used in our study was administrative data collected by English state-funded schools (the National Pupil Database – NPD) and hospitals (Hospital Episode Statistics – HES) using a national opt-out model for secondary usage (e.g., research and planning). The legal basis behind accessing this data was “public task” under Article 6 of the General Data Protection Regulations. Sharing this secure data for the purposes of this study via the Office of National Statistics Secure Research Service was done in line with the Digital Economy Act 2017. Permissions to use these linked, de-identified data under our legal basis from Hospital Episode Statistics and the National Pupil Database were reviewed and granted by the NHS Digital (DARS-NIC-381972) and the Department for Education (DR200604.02B) review boards respectively. Ethical approval for the ECHILD project that links HES and NPD was granted by the National Research Ethics Service (17/LO/1494), NHS Health Research Authority Research Ethics Committee (20/EE/0180) and UCL Great Ormond Street Institute of Child Health’s Joint Research and Development Office (20PE16) review boards. Consent from patients is not required for HES as the nationally collected administrative data is lawfully provided by NHS Digital in a pseudo-anonymised format that reduces identifiability to researchers; further information on opting out of Hospital Episode Statistics for secondary usage can be found here. Consequently, researchers had no access to information that could identify individual participants during or after data collection.

### Protocol

A protocol that outlines this project has previously been published [6]. One major difference since protocol development is the lack of instrumental variable and difference-in-difference (D-in-D) analysis. This is because we failed to find an appropriate instrument while the required parallel trend assumption, we would have needed to invoke to study hospitalisation rates using D-in-D was not met.

### Stakeholder involvement

Stake holder groups consisting of focus groups of young people, parents and service providers helped us frame the research question, interpret, and communicate our findings to policy makers, health and education services and families to promote translation of our findings into practice.

Prior to this study, two independent meetings were conducted with stakeholders (parents, pupils, teachers) to understand which medical conditions are of interest and which entry timepoints are important for child development. The first meeting was with the Department for Education’s national young SEN advisory group (the Friendship, Learning, Achieve, Reach and Empower group) on the 18 of September 2021 and the second with the Young Persons Advisory Group for research at Great Ormond Street Hospital on the 27 of November 2021. This engagement identified that school entry is an important key milestone when SEN provisions are required. Therefore, in the proposed study, we have used school start as our entry time for the assessment of eligibility, exposure status, and start of follow-up.

### Dataset and linkage

The data source was the Education and Child Health Insights from Linked Data (ECHILD) database [7], a pseudo-anonymised dataset that links the National Pupil Database (NPD) with Hospital Episode Statistics (HES), with a linkage rate of 95% [8]. This data was access on the 8^th^ February 2024. Currently follow-up of state-funded school and hospital activity is up to age 25 years (from birth in 1995 until hospitalisations in 2020). Its creation is described by Mc Grath-Lone *et al.*, 2021.In brief, the ECHILD’s extract of the NPD contains data from academic terms (October, January, and May) between 2006 and 2020 and contains information on (but not limited to) school, local authority (LA) of child’s home address and LA of school address, year/month of birth, gender, ethnicity, first language, socioeconomic status, free school meal status, absence related data, social care/children in need related data, key stage results, and SEN provision. The ECHILD’s extract of HES contains details on accident and emergency attendance, admitted patient care, critical care, and outpatient appointments between 1997 until 2021. In addition, it holds data on birth admissions, sex recorded by physician, ethnicity, clinical information recorded during hospital admissions, including details of diagnoses, and operations. HES covers 99% of public hospital activity in England (Herbert *et al.*, 2017) and, since 1998, is linked to ONS Mortality data covering information on causes and timing of deaths (Mc Grath-Lone *et al.* 2021).

### Study design and setting

This is a longitudinal observational study based on data from the ECHILD dataset which includes individuals born in an NHS funded hospital between 1 September 2003 and 31 August 2013 in England [7] and who were enrolled in Year 1 of primary education between 2008/2009 and 2018/2019. To reduce confounding-by-indication and other forms of biases when using observational data to address causal questions, we applied a target trial framework to guide our data extraction and definitions with regards to: eligibility, entry time, follow-up period, and to specify causal contrasts and their estimation [9]; details below). Analyses were conducted in the Office for National Statistics Secure Research Service using Stata ver. 17 (proprietary, StataCorp) and R ver. 4.0.2 (open source, R Foundation). Code is available https://github.com/UCL-CHIG/HOPE_CLP.

### Population

Our population consisted of children with CLP, but no other major congenital anomaly identified via HES records before age 5 year and who were born in NHS-funded hospital, linked to NPD from Year 1 of mainstream primary school (the first full year of compulsory education, where pupils are five years old on the first day) between academic years 2008/2009 and 2018/2019. The children included in the study were therefore born between 1 September 2003 and 31 August 2013. Exclusion of children with other major congenital abnormalities was chosen to reduce heterogeneity of needs for SEN provision. To classify pupils with CLP, the International Classification of Diseases version 10 (ICD-10) codes was applied to HES diagnoses in any hospital admission prior to the start of compulsory education using the following codes: Q35*, Q36* and Q37* [10]. For each pupil, the earliest recorded date in HES was considered the first phenotypical recording. Pupils whose first recording of CLP in HES was after their first year in school were not included to avoid instances whereby CLP diagnosis might be a consequence of SEN provision. Further major congenital anomalies prior to the start of follow-up were identified in hospital admission records using the EUROCAT code list [11] which captures major (and not minor) congenital anomalies, and excluded using the ICD-10 codes listed in S1 Table.

### Intervention

The causal question we wished to address concerns the impact of (recorded) SEN provision in Year 1 of primary school. SEN provision in the English educational setting is divided into two main categories: SEN Support (previously known as Action, Action Plus or non-Statemented SEN) and a more intensive Education and Health Care Plan (EHCP, this is previously known as a statement of SEN) [12]. SEN Support is organised by the educational environment (e.g., school or college) and provides help to children and young people in need of SEN provision, with support that may include teaching assistants who aid in communication, specialised adapted learning programmes and supporting physical needs. An EHCP is arranged and partially funded by local authorities for children and young people who require further adjustments and often require additional health specific resources (compared to SEN Support) to aid in education, health, and social care needs.

School-recorded SEN provision was categorised as having one of 3 categories: None, SEN Support, or Education and Health Care Plan (EHCP). Whilst SEN provision can change throughout a child’s educational journey, we focused on an intention-to-treat (ITT) effect of initiation of SEN provision, that is on the effect of SEN provision recorded at the start of compulsory education (and not whether this was adhered to during primary education). Hence, our causal question relates to the impact of assignment to a specific category of SEN provision at the start of compulsory education, and not to whether assignment was planned to be continued and adhered to. To establish SEN status, we used the January (Spring) census in Year 1 of primary education because school funding is calculated from these data (S2 Table) and therefore they are more complete. We focus on recorded SEN assignment as we have no access to whether (and how) SEN intervention was provided.

### Follow-up

Pupils were followed-up from the January census in Year 1 until the end of primary school (end of July of Year 6), loss to follow-up, or end of study, whichever occurred first. Children were considered lost to follow-up if they no longer appear in any NPD school census during primary education; this could be due to a variety of reasons including transfer to a non-state-funded school, home schooling, emigration, death, or off-rolling [13].

### Outcome variables

The outcomes of interest were unplanned hospital usage, school absences and educational attainment.

Our unplanned hospital usage is a combination of accident and emergency usage and admitted patient care. To identify unplanned hospital utilisation in HES Admitted Patient Care, we used the “admission method” variable of the first episode of each admission in HES (admimeth) (S3 Table). We used the HES Accident and Emergency dataset to account for non-admitted unplanned hospital utilisation that did not require an overnight stay [14]. We combined the “Admitted Patient Care” and “Accident and Emergency” datasets to create the cumulative number of days of unplanned hospital usage between the January census in Year 1 and the end of follow-up. When an unplanned admission and recording in accident and emergency occurred on the same day, we only counted this as a single day hospital utilisation, for example when the pupil is initially presented in accident and emergency and is then admitted on the same day. Rates of unplanned hospital usage were then calculated by dividing the cumulative number of days of unplanned hospitalisation by the total follow-up (also measured in days).Values are then reported as yearly rates.

Absences in NPD are recorded termly as number of missed sessions, corresponding to half-days in school. Cumulative number of school absences were therefore calculated as number of half-day sessions between January of Year 1 and the end of follow up. The total number of potential sessions in a term is also available and thus rates of absences were derived as the ratio of missed sessions over potential sessions. NPD holds information on type of absences (authorised and unauthorised, each also subdivided into subgroups, e.g., medical absences, authorised vacation and unauthorised vacation; S4 Table). Rates of medical and unauthorised absences were then calculated by dividing the cumulative number of missed half-day sessions by the total number of half-day sessions. Furthermore, in line with government measures, we’ve also evaluated persistent absences, defined as 10% or more absence sessions during the follow up.

The third set of outcomes of interest included the academic performance in the summer term of Year 2 and Year 6, Key Stage 1 (KS1) and Key Stage 2 (KS2) mathematics scores and their progress relative to school readiness (EYFSP). We choose mathematics scores as these scores are externally validated and are considered more objective when compared to other assessment subjects such as English. They have also been shown to better predict later Maths and English outcomes than English at KS2 [15].The original scores were standardised across the whole NPD database separately by academic year to account for marking variations.

### Covariates

To account for non-random SEN provision assignment, we used information on several covariates that are known or suspected to confound the association between SEN provision and our outcomes of interest. These include birth, socio-demographic, and clinical features of the child as well as geographical and school characteristics. These are listed in Table 1 with their presumed relationships depicted in S1 Fig. Broadly, these covariates include: cleft severity [16], chronic conditions (categorised as in [17]), gender, gestational age, maternal age, birth weight, month of birth, racial-ethnic group (latest recorded in NPD to reduce missingness), English as a first language, academic cohort, rate of prior hospital contact (planned and unplanned, definitions in S3 Table), Income Deprivation Affecting Children Index (IDACI; in quintiles, recorded in NPD according to the child’s address postal code), LA of the child’s home address, free school meal eligibility, and school early assessment (Early Years Foundation School Profile, EYFSP) z-score. EYFSP is available only for children who attended school (“Reception Class”) before they started compulsory education in year 1. School-level variables were the proportion of children with SEN provision during the previous academic year (“historical SEN provision”), the proportion of children entitled to receive free school meal (“historical FSM eligibility”), and whether the school had nursery classes.

**Table 1:** Description of selected pupil characteristics by categories of Special Education Needs provision in Year One.

	Special Education Needs Provision			
Characteristics	None (N = 4350, 65.9%)	Special education needs Support (N = 2009, 30.4%)	Education and Healthcare Plan (N = 242, 3.7%)	Total (N = 6601, 100%)
**Birth characteristics**				
**Gender**				
Female	1949 (71.5%)	709 (26.0%)	68 (2.5%)	2726 (100.0%)
Male	2401 (62.0%)	1300 (33.5%)	174 (4.5%)	3875 (100.0%)
**Gestational Age (Weeks)**				
34 Weeks or Less	91 (49.2%)	75 (40.5%)	19 (10.3%)	185 (100.0%)
35-36 Weeks	149 (62.3%)	80 (33.5%)	10 (4.2%)	239 (100.0%)
37-38 Weeks	664 (63.4%)	346 (33.0%)	37 (3.5%)	1047 (100.0%)
39 Weeks+	2525 (69.6%)	993 (27.4%)	112 (3.1%)	3630 (100.0%)
Unknown	921 (61.4%)	515 (34.3%)	64 (4.3%)	1500 (100.0%)
**Birthweight Category**				
2500g-3499g	1920 (65.4%)	901 (30.7%)	114 (3.9%)	2935 (100.0%)
3500g and higher	1493 (72.2%)	531 (25.7%)	44 (2.1%)	2068 (100.0%)
Less than 2500g	270 (54.1%)	191 (38.3%)	38 (7.6%)	499 (100.0%)
Unknown	667 (60.7%)	386 (35.1%)	46 (4.2%)	1099 (100.0%)
**Maternal Age (Years)**				
<20	266 (57.3%)	185 (39.9%)	13 (2.8%)	464 (100.0%)
20-24	820 (61.2%)	482 (36.0%)	37 (2.8%)	1339 (100.0%)
25-29	1161 (66.4%)	522 (29.8%)	66 (3.8%)	1749 (100.0%)
30-34	1168 (70.2%)	431 (25.9%)	65 (3.9%)	1664 (100.0%)
35 or higher	770 (68.6%)	306 (27.2%)	47 (4.2%)	1123 (100.0%)
Unknown	165 (63.0%)	83 (31.7%)	14 (5.3%)	262 (100.0%)
**Demographics**				
**Ethnic Group (latest in the national pupil database)**				
Recorded as White	3646 (66.2%)	1669 (30.3%)	192 (3.5%)	5507 (100.0%)
Not recorded as White	704 (64.4%)	340 (31.1%)	50 (4.6%)	1094 (100.0%)
**Language Group**				
Recorded as English	3796 (66.0%)	1750 (30.4%)	206 (3.6%)	5752 (100.0%)
Not recorded as English	554 (65.3%)	259 (30.5%)	36 (4.2%)	849 (100.0%)
Unknown				
**Income Deprivation Affecting Children Index Quintile**				
(Most deprived) 1	983 (56.6%)	703 (40.4%)	52 (3.0%)	1738 (100.0%)
2	913 (63.8%)	468 (32.7%)	51 (3.6%)	1432 (100.0%)
3	861 (67.4%)	360 (28.2%)	57 (4.5%)	1278 (100.0%)
4	817 (72.6%)	266 (23.6%)	42 (3.7%)	1125 (100.0%)
(Least deprived) 5	768 (75.7%)	207 (20.4%)	39 (3.8%)	1014 (100.0%)
Unknown	8 (57.1%)	5 (35.7%)	1 (7.1%)	14 (100.0%)
**Free School Meal Eligibility**				
Not Eligible	3706 (70.0%)	1403 (26.5%)	189 (3.6%)	5298 (100.0%)
Eligible	644 (49.4%)	606 (46.5%)	53 (4.1%)	1303 (100.0%)
**Academic Year**				
2008/2009	306 (61.1%)	173 (34.5%)	22 (4.4%)	501 (100.0%)
2009/2010	332 (60.4%)	199 (36.2%)	19 (3.5%)	550 (100.0%)
2010/2011	346 (62.7%)	185 (33.5%)	21 (3.8%)	552 (100.0%)
2011/2012	348 (61.3%)	197 (34.7%)	23 (4.0%)	568 (100.0%)
2012/2013	384 (64.4%)	186 (31.2%)	26 (4.4%)	596 (100.0%)
2013/2014	415 (66.7%)	190 (30.5%)	17 (2.7%)	622 (100.0%)
2014/2015	374 (67.0%)	159 (28.5%)	25 (4.5%)	558 (100.0%)
2015/2016	440 (68.9%)	179 (28.0%)	20 (3.1%)	639 (100.0%)
2016/2017	463 (69.6%)	176 (26.5%)	26 (3.9%)	665 (100.0%)
2017/2018	463 (70.8%)	171 (26.1%)	20 (3.1%)	654 (100.0%)
2018/2019	479 (68.8%)	194 (27.9%)	23 (3.3%)	696 (100.0%)
**Clinical**				
**Type of Cleft**				
Cleft Lip only	1284 (78.6%)	319 (19.5%)	30 (1.8%)	1633 (100.0%)
Cleft Palate only	1852 (64.2%)	892 (30.9%)	141 (4.9%)	2885 (100.0%)
Unilateral CLP	980 (59.5%)	619 (37.6%)	49 (3.0%)	1648 (100.0%)
Bilateral CLP	234 (53.8%)	179 (41.1%)	22 (5.1%)	435 (100.0%)
**Chronic Condition – Any**				
No	2835 (74.7%)	908 (23.9%)	51 (1.3%)	3794 (100.0%)
Yes	1515 (54.0%)	1101 (39.2%)	191 (6.8%)	2807 (100.0%)
**Chronic Condition – Blood Cancer**				
No	4290 (66.3%)	1958 (30.3%)	224 (3.5%)	6472 (100.0%)
Yes	60 (46.5%)	51 (39.5%)	18 (14.0%)	129 (100.0%)
**Chronic Condition –Mental Health Behaviour**				
No	4297 (67.7%)	1883 (29.7%)	167 (2.6%)	6347 (100.0%)
Yes	53 (20.9%)	126 (49.6%)	75 (29.5%)	254 (100.0%)
**Chronic Condition – Endocrine Digestive Renal Genitourinary**				
No	4143 (67.1%)	1846 (29.9%)	189 (3.1%)	6178 (100.0%)
Yes	207 (48.9%)	163 (38.5%)	53 (12.5%)	423 (100.0%)
**Chronic Condition – Non-specific Codes**				
No	4211 (68.5%)	1795 (29.2%)	145 (2.4%)	6151 (100.0%)
Yes	139 (30.9%)	214 (47.6%)	97 (21.6%)	450 (100.0%)
**School-related**				
**Early Years Foundation Profile – All – (z-score)**				
Median	0.0	−0.8	−2.1	−0.1
Q1, Q3	−0.3, 0.6	−1.6, −0.1	−2.3, *	−0.9, 0.3
Missing	32 (54.5%)	20 (32.7%)	9 (14.8%)	61 (100%)
**Relative Age**				
5 years and 0 months old	341 (60.2%)	205 (36.2%)	20 (3.5%)	566 (100.0%)
5 years and 1 months old	383 (61.9%)	215 (34.7%)	21 (3.4%)	619 (100.0%)
5 years and 2 months old	339 (61.6%)	188 (34.2%)	23 (4.2%)	550 (100.0%)
5 years and 3 months old	384 (64.4%)	189 (31.7%)	23 (3.9%)	596 (100.0%)
5 years and 4 months old	340 (64.3%)	173 (32.7%)	16 (3.0%)	529 (100.0%)
5 years and 5 months old	322 (66.9%)	141 (29.3%)	18 (3.7%)	481 (100.0%)
5 years and 6 months old	338 (70.4%)	126 (26.2%)	16 (3.3%)	480 (100.0%)
5 years and 7 months old	388 (68.4%)	156 (27.5%)	23 (4.1%)	567 (100.0%)
5 years and 8 months old	354 (63.1%)	182 (32.4%)	25 (4.5%)	561 (100.0%)
5 years and 9 months old	392 (71.5%)	140 (25.5%)	16 (2.9%)	548 (100.0%)
5 years and 10 months old	383 (69.1%)	148 (26.7%)	23 (4.2%)	554 (100.0%)
5 years and 11 months old	386 (70.2%)	146 (26.5%)	18 (3.3%)	550 (100.0%)

### Target trial emulation

To reduce confounding and other sources of bias likely to affect analyses of observational data, we adopted a Target Trial Emulation (TTE) framework [9]. TTE enables observational data to be mapped to a hypothetical target experimental trial counterpart by creating the specification of an ideal (pragmatic) trial and using this as a basis to shape the observational study design. TTE consists of: (a) defining the specifications of a hypothetical target experimental trial of the causal question of interest (including the corresponding causal contrast); (b) emulating the specifications of the ideal target trial using observational data; and (c) estimating the effects of interest using the emulated trial data. The first component of TTE involves defining inclusion/exclusion criteria for entry, a treatment strategy (including time of treatment assignment), follow-up frequency and modality, outcome measures, causal contrasts of interest (“estimands”) and estimation methods. The second component of TTE involves handling the observational data to emulate the structure of the data that would be gathered in the specified target trial. Finally, the third component of TTE concerns dealing with the inevitable confounding that affects observational data and explicitly outlining the analytical methodology ahead of the data wrangling. S5 Table outlines the protocols for the ideal and emulated target trials designed for this study.

To investigate the causal effect of SEN provision in Year 1 for children with isolated CLP on our health and educational outcomes we targeted two causal contrasts: the average treatment effect (ATE) and the average treatment effect on the treated (ATT) [18]. The ATE is a causal contrast of average potential outcomes for the whole study population and therefore captures the marginal causal effect for all CLP children without further MCAs; the ATT is a causal contrast restricted to the “treated”, i.e., those that were assigned to the SEN intervention, thus capturing the impact of removing the assignment in the subset of CLP children (without further MCAs) who actually had been assigned it. Because the intervention has three categories (None, SEN Support, and EHCP), we defined these causal contrasts as comparisons of respectively: (i) SEN Support versus None, (ii) EHCP versus None, and (iii) EHCP versus SEN Support.

### Analysis

#### Representativeness.

We compared the proportions of children with CLP without further MCAs in ECHILD who were in Year 1 between 2008/09 and 2018/19 with proportions published for children with CLP born in England [19]; we also compared their average KS1 scores among them against published results for the same population of children [16].

#### Descriptive statistics.

We examined the distribution of each confounder and each outcome by categories of SEN provision to gather some evidence of associations. We also examined the patterns of missing values observed in the covariates by fitting separate logistic regression models for the indicator of missingness created for each covariate; models included all other variables with complete records.

#### Causal effects.

The targeted causal contrasts (ATE and ATT) were expressed as rate ratios (RaR) for hospitalisation and absence rates; as risk ratios (RiR) for persistent absences; and as mean score differences (Δ) for KS1 and KS2. Their estimation was carried out using related approaches that rely on slightly different parametric assumptions: inverse probability weighting (IPW) by propensity score (PS), g-computation, and augmented inverse probability weighting (AIPW) [20]. Each of these approaches invokes the assumptions of no interference, consistency and no unmeasured confounding; however, they differ in terms of the relationships that are modelled to deal with confounding [21]. Specifically, IPW requires that the relationship between exposure and confounders (the propensity score model) is correctly specified; AIPW that either the outcome or the propensity score model is correctly specified (a double robustness property); g-computation that the outcome model is correctly specified. IPW and AIPW also invoke the assumption of positivity, i.e., that each member of the population has a non-zero chance of receiving each level of the exposure, conditional on confounders.

To implement estimation by IPW and AIPW, the association between the exposure (recorded SEN provision in Year 1) and the identified confounders was examined by fitting three logistic regression models (“propensity score (PS) models”) for: (i) SEN Support versus None, (ii) EHCP versus None; and (iii) EHCP versus SEN Support. In each case only children observed to have been assigned to one of the categories being compared were included in the analyses. PS were predicted from each of these models. When implementing IPW continuous confounders were modelled using linear and quadratic terms. The model-specific PS distributions were graphically plotted separately by category of exposure to assess whether distributions overlapped and hence whether the study participants had non-zero probabilities of being “exposed” at each level of the PS (i.e., whether there was support for the assumption of positivity). When implementing AIPW, the least absolute shrinkage and selection operator (Lasso) was used to optimise the parametric specification of both the outcome and PS model [22]].

With each of IPW, AIPW and g-computation, outcome models were specified as follows: for unplanned hospital utilisation, medical and unauthorised absences, Negative Binomial models were used to reflect the extra dispersion in the data (but Poisson models were used with AIPW due to computing constraints); for persistent absences a logistic regression model was used; and for KS1 and KS2 linear regression models. We dealt with the clustering of pupils within the child’s home LA by reporting either robust standard errors (SEs) or bootstrap SEs that reflected the clustering (with 1000 bootstraps).

Triangulating the results obtained by these three approaches offered insights on their robustness and possible sources of bias. Additionally, for reference, we report the results obtained via standard univariable and multivariable regression for each outcome, with the latter leading to estimates of conditional effects.

#### Missing data.

Missing data in the confounders were addressed using the missing covariate indicator method (MCIM) which is widely used in both econometrics and applications involving electronic health records [23]. It consists of creating a dummy variable indicating missingness for a given covariate while the value of that covariate is set to zero when missing, with both variables included in the model, as relevant. Although this approach is known to suffer from residual confounding [24,25], simulations by Song et al [26] showed that MCIM leads to introduce a very small bias in scenarios typical of epidemiological studies.

#### Sensitivity analyses.

To examine whether time varying effects may affect the results, we re-run the analyses censoring follow-up at the end of Year 2 (as opposed to Year 6). We also rerun the analyses including only records with complete confounder information (as opposed to using the MCIM approach). Finally, we disaggregated hospitalisation usage to “accident and emergency” usage and “admitted patient care” due to their differential pathways with “admitted patient care” requiring a physician to admit the patient.

## Results

### Study population

**Fig 1:**
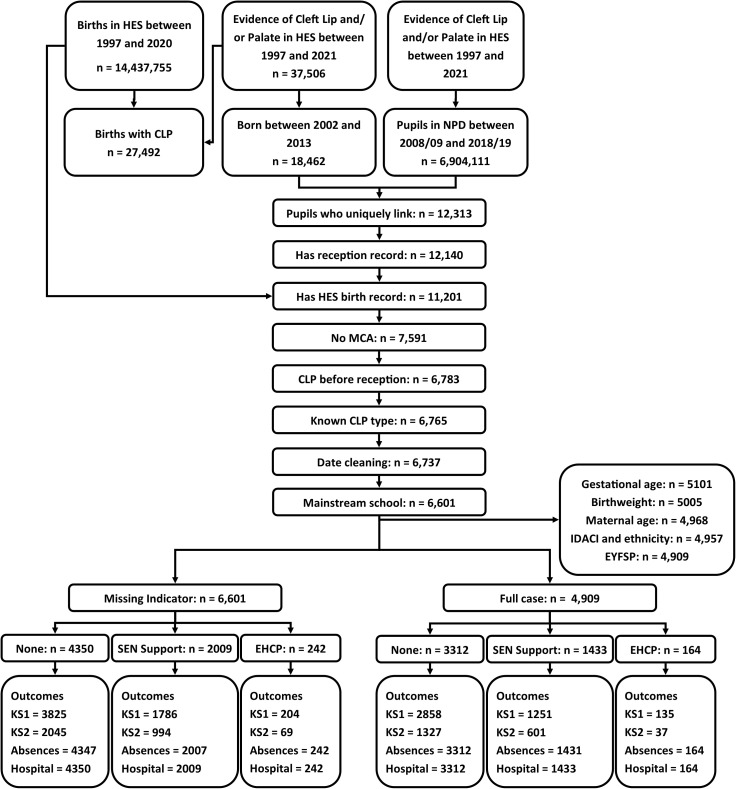
Diagram depicting cohort derivation. This figure depicts the derivation of the cohort starting from combining births in hospital episode statistics with evidence of cleft lip and/or palate between 2003 and 2013 who are linkable to the national pupil database between 2008/2009 and 2018/2019. Exclusion criteria were: pupils with multiple links between hospital episode statistics and the national pupil database, had no record in reception class, has no birth record in hospital episode statistics, has a recorded major congenital anomaly in hospital episode statistics, a first phenotypical recording of cleft lip and/or palate after reception, an unknown cleft lip and/or palate type, conflicting date information (e.g., linked mortality data states a death before school start), and a recorded attendance at a special school.

We found a total of 37,506 unique patients in HES Admitted Patient Care between 1997 and 2021 who had at least one phenotypical recording for Cleft Lip and/or Palate. Linkage with NPD records from 2008/2009–2018/2019 Spring terms, identified 11,201 children who had birth records in HES and Reception in NPD (the “source population”). Of these, 6,783 children had a recorded CLP diagnosis before start of compulsory schooling (Year 1) and no additional major congenital anomalies. We excluded 46 children without specific information CLP subtype information and further 136 who did not attend mainstream school in Year 1 (and thus did not have information on school attendance and performance), leading to the study population of 6,601 isolated CLP children (Fig 1).

### Representativeness

The rate of Cleft Lip and/or Palate among the births between 1997–2020 children recorded in ECHILD was 19 per 10,000 births, which was greater than the rate found among children contributing to the CRANE registry (15 CLP patients per 10,000 births between 2009 and 2018. See S6 Table for a comparison of CLP rates through time. The distribution of CLP types in our population and in CRANE was nevertheless similar [19] (See S7 Table) as were the rates of comorbidities (38% versus 36%; [27]). Furthermore, the patterns of the EYFSP z-scores for mathematics were similar to those previously published [4] (See S2 Fig)

### Descriptive statistics

Of the 6,601 pupils included in our study population, 30.4% were recorded to have SEN Support and 3.7% to EHCP in the Year One January Census (Table 1).There were proportionally more SEN Support and EHCP recordings among boys than girls, and among those born prematurely and at <2500g in weight than in the other categories; they were also more prevalent among those not recorded as White, not with English as primary language and those eligible for free school meals, relative to their counterparts. In contrast, there were proportionally fewer EHCP and more SEN Support recordings among those living in more deprived areas, eligible to free school meals and with younger mothers (Table 1). The distribution of CLP type and chronic conditions showed that children without recorded SEN Support were more likely to have less severe CLP (i.e., Cleft lip only) and mostly not to have any chronic conditions; in contrast children recorded to have been assigned to EHCP in Year 1 were more likely to have cleft palate only or bilateral CLP, to have mental health behaviour and non-specific conditions. The average EYFSP score decreases incrementally with type of SEN support (Table 1).

The numbers and rates of unplanned hospital utilization, medical and unauthorised absences, and academic performance by the end of Year 6 are shown in Table 2*.* Overall, the rates of unplanned hospitalisation were 384.8 per 1,000 years and the rates of medical and unauthorised absences were 35.3 and 9.8 per 1,000 sessions. Persistent absences were observed in 10.1% of the pupils. The rates of unplanned hospital utilisation and of medical absences increase with increasing intensity of SEN provision, with the percentages of persistent absences following the same pattern, while rates of unauthorised absences are higher for SEN Support than for EHCP children, and both are higher than for no SEN provision children (Table 2).

**Table 2:** Description of the study outcomes by categories of SEN provision in Year 1^(a)^.

		Special Education Needs Provision			
Outcome		None (N = 4350, 65.9%)	Special Education Needs Support (N = 2009, 30.4%)	Education and Healthcare Plan (N = 242, 3.7%)	Total (N = 6601, 100%)
**Unplanned Hospital Utilisation**					
	**N**	**4350**	**2009**	**242**	**6601**
	Total number of days in hospital	5634	3608	642	9884
	Total number of follow up Years	16,931.2	8,276.6	994.9	26,202.7
	Rate 1,000 years	***336.7***	***439.3***	***796.9***	***384.8***
**Medical Absences**					
	**N**	4347	2007	242	6596
	Total number of sessions	195,102	127,338	18,938	341,378
	Total number of possible sessions	6,277,697	3,028,397	341,017	9,647,111
	Rate per 1000 sessions	***31.0***	***42.0***	***58.1***	***35.3***
**Unauthorized Absences**					
	**N**	4347	2007	242	6596
	Total number of sessions	50,470	33,514	3,279	87,263
	Total number of possible sessions	6,277,697	3,028,397	341,017	3,471,111
	Rate per 1000 sessions	***9.1***	***11.2***	***10.4***	***9.8***
**Persistent Absences** ^**(b)**^					
	**N**	**4347**	**2007**	**242**	**6596**
	Number pupils	314	295	54	663
	Percentage	7.2%	14.7%	22.3%	10.1%
**Key Stage 1 Math Score** ^**(c)**^					
	N	**3825**	**1786**	**204**	**5815**
	**Median**	0.1	−0.7	−1.57	0.1
	(25^th^, 75^th^ centile)	−0.3, 0.3	−1.2, 0.1	−2.38, -	−0.8, 0.3
**Early Years Foundation Profile to Key Stage 1 math progression** ^**(c)**^					
	N	3795	1768	196	5759
	**Median**	**0.1**	**0.1**	**–**	**0.1**
	(25^th^, 75^th^ centile)	−0.6, 0.5	−0.5, 0.64	−0.2, 0.825	−0.5, 0.55
**Key Stage 2 Math Score** ^**(c)**^					
	N	2045	994	69	3108
	**Median**	**0.25**	**−0.7**	**–**	**0.0**
	(25^th^, 75^th^ centile)	−0.5, 0.8	−1.7, 0.2	-,-	−0.9, 0.7
**Early Years Foundation Profile to Key Stage 2 Math Progression** ^**(c)**^					
	N	2030	985	–	3082
	Median	0.0	0.0	–	0.0
	(25^th^, 75^th^ centile)	−0.6, 0.5	−0.8, 0.7	-,-	−0.7, 0.6

^(^a^)^Total numbers vary because of missing absence data or varying follow-up (for Key Stage results)

^(^b^)^More than 10% absences overall between Year 1 and Year 6.

^(^c^)^Numbers are reduced because not all children in the study population reached the age when the tests are carried out

KS1 (N = 5,815) and KS2 (N = 3,108) mathematics scores of the children who reached the relevant ages and sat the examinations were on average negative (median = −0.10, IQR: −0.8,0.3; median = −0.21, SD = 1.10, respectively). Progression from pre-school levels (EYFSP) was generally null (Table 2).

**Table 3:** Estimated causal rate ratios (RaR) and causal risk ratios (RiR) of the effect of Special Education Needs Support versus No provision for unplanned hospitalization and absences by estimation method.

Outcome	Exposure	N	(%)	Causal contrast	Estimation Method							
					Regression		Inverse Probability Weighting		G-computation		Augmented Inverse Probability Weighting^(a)^	
					RaR	95% CI	RaR	95% CI^(b)^	RaR	95% CI^(b)^	RaR	95% CI^(b)^
**Unplanned Hospital Utilization**	**All**	**6,359**	(100.0)	Crude Ass.	**1.31**	1.12, 1.52	**–**	–	**–**	–	**–**	–
	No provision	4,350	(68.4)	Cond. Ass.	**0.99**	0.90, 1.09	**–**	–	**–**	–	**–**	–
	Special Education Needs Support	2,009	(31.6)	ATE	**–**	–	**1.16**	1.00, 1.34	**0.99**	0.87, 1.12	**1.02**	0.87, 1.17
				ATT	**–**	–	**1.10**	0.92, 1.33	**0.95**	0.79, 1.10	**0.98**	0.79, 1.17
**Medical Absences**	**All**	**6,354**	(100.0)	Crude Ass.	**1.34**	1.28, 1.40	**–**	–	**–**	–	**–**	–
	No provision	4,347	(68.4)	Cond. Ass.	**1.10**	1.05, 1.15	**–**	–	**–**	–	**–**	–
	Special Education Needs Support	2,007	(31.6)	ATE	**–**	–	**1.10**	1.04, 1.18	**1.09**	1.03, 1.15	**1.04**	0.95, 1.13
				ATT	**–**	–	**1.07**	0.99, 1.15	**1.05**	0.98, 1.12	**1.04**	0.97, 1.10
**Unauthorised Absences**	**All**	**6,354**	(100.0)	Crude Ass.	**1.22**	1.11, 1.34	**–**	–	**–**	–	**–**	–
	No provision	4,347	(68.4)	Cond. Ass.	**0.96**	0.87, 1.07	**–**	–	**–**	–	**–**	–
	Special Education Needs Support	2,007	(31.6)	ATE	**–**	–	**0.86**	0.76, 0.97	**0.98**	0.86, 1.09	**0.80**	0.66, 0.95
				ATT	**–**	–	**0.89**	0.75, 1.07	**0.99**	0.84, 1.13	**0.79**	0.62, 0.96
					OR	95% CI	RiR	95% CI^**(b)**^	RiR	95% CI^**(b)**^	RiR	95% CI
**Persistent Absences** ^(c)^	**All**	**6,354**	(100.0)	Crude Ass.	**2.21**	1.87, 2.62	**–**	–	**–**	–	**–**	–
	No provision	4,347	(68.4)	Cond. Ass.	**1.13**	0.92, 1.39	**–**	–	**–**	–	**–**	–
	Special Education Needs Support	2,007	(31.6)	ATE	**–**	–	**1.13**	0.92, 1.34	**1.08**	0.86, 1.31	**1.20**	0.96, 1.45
				ATT	**–**	–	**1.12**	0.88, 1.36	**1.12**	0.88, 1.36	NA	NA

ECHILD cohort of isolated cleft lip and/or palate born in NHS England hospitals between 2003 and 2013. Estimates replicated in R and Stata, except for those by Augmented Inverse Probability Weighting that were obtained only in Stata. Confidence intervals (CI) were calculated using

^(^b^)^1000 bootstraps and account for clustering by home address local authority.

^(^a^)^Fitted using a Poisson outcome regression model as opposed to Negative Binomial. RaR: rate ratio; RiR: risk ratio; Ass: association; Cond: conditional; ATE: average treatment Effect; ATT: Average Treatment Effect in the treated.

#### Causal effects.

**Fig 2:**
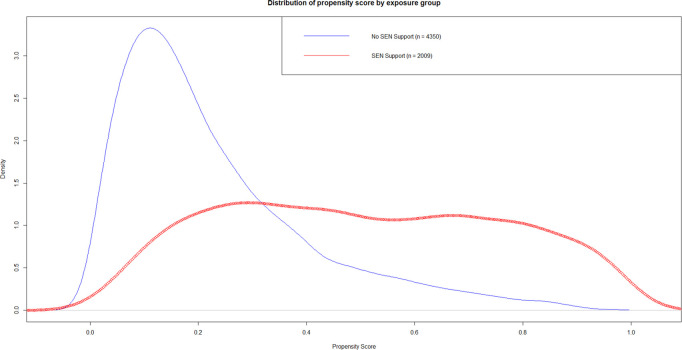
Predicted propensity score distributions by observed special educational needs category for Special Educational Needs Support versus None. This diagram depicts the density distribution of the probability of receiving SEN Support in reference to None. Predictors used to estimate treatment probability included: gender, gestational age, birthweight category, maternal age, ethnic group, language group, income deprivation affecting children index quintile, free school meal eligibility, academic year, type of cleft lip and/or palate, chronic conditions, early years foundation profile z-score and relative age.

**Fig 3:**
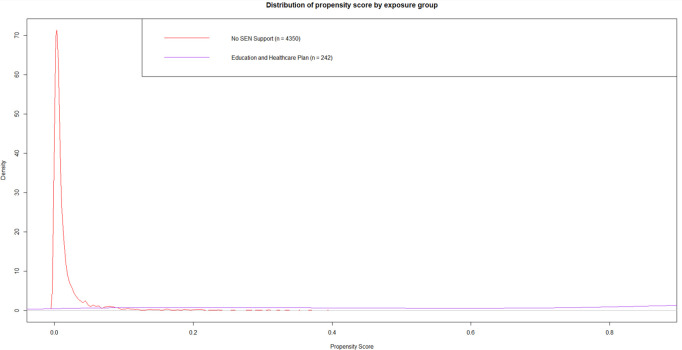
Predicted propensity score distribution by observed special educational needs category for Education and Healthcare Plan versus None. This diagram depicts the density distribution of the probability of receiving an Education and Healthcare Plan in reference to None. Predictors used to estimate treatment probability included: gender, gestational age, birthweight category, maternal age, ethnic group, language group, income deprivation affecting children index quintile, free school meal eligibility, academic year, type of cleft lip and/or palate, chronic conditions, early years foundation profile z-score.

**Fig 4:**
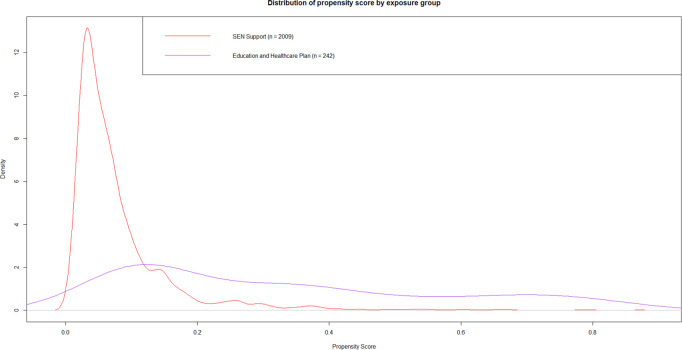
Predicted propensity score distribution by observed special educational needs category for Education and Healthcare Plan versus Special Education Needs Support. This diagram depicts the density distribution of the probability of receiving an Education and Healthcare Plan in reference to Special Education Needs Support. Predictors used to estimate treatment probability included: gender, gestational age, birthweight category, maternal age, ethnic group, language group, income deprivation affecting children index quintile, free school meal eligibility, academic year, type of cleft lip and/or palate, chronic conditions, early years foundation profile z-score.

Three PS models were fitted to predict the propensity of being assigned to, respectively: (i) SEN Support or None, (ii) EHCP or None; and (iii) EHCP or SEN Support. The distribution of the predicted PS for each of these models revealed that there was good overlap for the comparison of (i) SEN Support versus None (Fig 2), but very poor overlap for the comparisons of (ii) EHCP versus None (Fig 3), and (iii) EHCP versus SEN Support (Fig 4). See S8 Table for a detailed tabulated breakdown of propensity scores categories by treatment category. Also see S3 Fig to S8 Fig for validation of the propensity scores using complementary machine learning methods. Therefore, for the rest of this paper we focus on (i) SEN Support vs. None only.

### Unplanned hospital utilisation

The greater rates in unplanned hospitalisation for children assigned to SEN Support relative to None, amounting to a 31% relative increase (RR_crude_ = 1.31, 95% confidence interval (CI): (1.12, 1.52), Table 3), was partly reduced when the ATE was estimated by IPW (RR_IPW_ = 1.16 (1.00,1.34)) and completely removed when estimated by g-computation or AIPW (RR_g_ = 0.99 (0.87, 1.12); RR_AIPW_ = 1.02 (0.87, 1.17)). When the hypothetical comparison of offering SEN Support or None was restricted to the children who actually were assigned to SEN Support, the estimated ATT indicated no effect of SEN Support when using g-computation or AIPW but possibly some effect when estimated using IPW (RR_ATT_g_ = 0.95 (0.79, 1.10); RR_ATT_AIPW_ = 0.98 (0.79, 1.17); RR_ATT_IPW_ = 1.10 (0.92, 1.33); Table 3).

### Absences

There was some evidence- although not supported by all estimation approaches- of an increase in the rates of medical absences associated with SEN Support relative to None, even after accounting for confounding (RR_crude_ = 1.34 (1.28, 1.40); RaR_IPW_ = 1.10 (1.04, 1.18); RaR_g_ = 1.09 (1.03, 1.15); RaR_AIPW_ = 1.04 (0.95, 1.13); Table 3). This was also found when examining the effect on the treated population (RR_ATT_IPW_ = 1.07 (0.99, 1.15); RR_ATT_g_ = 1.05 (0.98, 1.12); RR_ATT_AIPW_ = 1.04 (0.97, 1.10); Table 3). In contrast, the apparent greater rates of unauthorised absences in children assigned to SEN Support relative to those assigned to None (RR_crude_ = 1.22 (1.11, 1.34)), were found to be explained by confounding, with the possibility of a protective effect, (RR_IPW_ = 0.86 (0.76, 0.97), RR_g_ = 0.98 (0.86, 1.09), and RR_AIPW_ = 0.80 (0.66, 0.95); similar results were found for the ATT (Table 3). Likewise, the apparent greater odds of persistent absences for children assigned to SEN Support relative to None (OR_crude_ = 2.21 (1.87, 2.62)) were reduced to showing no effect both for the overall and the treated population when confounding was accounted for (RiR_ATE_IPW_ = 1.13 (0.92, 1.34); RiR_ATE_g_ = 1.08 (0.86, 1.31); RiR_ATE_AIPW_ = 1.20 (0.96, 1.45) and similarly for the ATT; Table 3).

#### Key Stage 1 and Key Stage 2 mathematics scores.

As already observed, children with CLP who have recorded SEN support have below average KS scores (KS1: Δ_crude_ = −0.85 (−0.90, −0.79); KS2: Δ_crude_ = −0.82 (−0.89, −0.75); Table 4); this apparent disadvantage is reduced but not completely when the ATE is estimated (for KS1: Δ_IPW_ = −0.18 (−0.25, −0.10); Δ_g_ = −0.21 (−0.26, −0.16); Δ_AIPW_ = −0.25 (−0.32, −0.17); and for KS2: Δ_IPW_ = −0.24 (−0.33, −0.15); Δ_g_ = −0.27 (−0.33, −0.21); Δ_AIPW_ = −0.23 (−0.32, −0.17); Table 4). These results are mirrored when the scores are replaced by their relative progress from reception results (the difference between KS scores and EYFSP scores) and when restricted to the treated population (Table 4).

**Table 4:** Estimated causal mean differences (Δ) of the effect of SEN Support versus No SEN provision for key stage test results by estimation method.

Outcome	Exposure	N	(%)	Causal contrast	Estimation Method							
					Regression		Inverse Probability Weighting		G-computation		Augmented Inverse Probability Weighting	
					Δ	95% CI	Δ	95% CI^(a)^	Δ	95% CI^(a)^	Δ	95% CI^(a)^
**Key Stage 1- Math**	**All**	**5611**	(100.0)	Crude Ass.	−0.85	−0.90, −0.79	**–**	–	**–**	–	**–**	–
	No provision	3,825	(68.2)	Cond. Ass.	−0.24	−0.29, −0.19	**–**	–	**–**	–	**–**	–
	Special Education Needs Support	1,786	(34.3)	ATE	–	–	−0.18	−0.25, −0.10	−0.21	−0.26, −0.16	**−0.25**	−0.32, −0.17
				ATT	**–**	–	−0.31	−0.40, −0.22	−0.30	−0.35, −0.24	**–**	–
**Key Stage 2- Math**	**All**	3039	(100.0)	Crude Ass.	−0.82	−0.89, −0.75	**–**	–	**–**	–	**–**	–
	No provision	2045	(67.3)	Cond. Ass.	−0.29	−0.36, −0.23	**–**	–	**–**	–	**–**	–
	Special Education Needs Support	994	(32.7)	ATE	–	–	−0.24	−0.33, −0.15	−0.27	−0.33, −0.21	**−0.23**	−0.31, −0.16
				ATT	**–**	–	−0.34	−0.45, −0.23	−0.32	−0.40, −0.24	**–**	–
**Early Years Foundation Stage Profile -Key Stage 1 progress- Math**	**All**	3027	(100.0)	Crude Ass.	0.07	0.02, 0.12	**–**	–	**–**	–	**–**	–
	No provision	2036	(67.3)	Cond. Ass.	−0.23	−0.28, −0.18	**–**	–	**–**	–	**–**	–
	Special Education Needs Support	991	(32.7)	ATE	–	–	−0.25	−0.35, −0.16	−0.21	−0.26, −0.16	**−0.24**	−0.32, −0.17
				ATT	**–**	–	−0.26	−0.34, −0.18	−0.27	−0.33, −0.22	**–**	–
**Early Years Foundation Stage Profile -Key Stage 2 progress- Math**	**All**	3015	(100.0)	Crude Ass.	**0.03**	−0.04, 0.10					**–**	–
	No provision	2030	(67.3)	Cond. Ass.	**−0.26**	−0.33, −0.19					**–**	–
	Special Education Needs Support	985	(32.7)	ATE			**−0.23**	−0.31, −0.14	**−0.26**	−0.32, −0.19	**−0.22**	−0.30, −0.15
				ATT			**−0.27**	−0.37, −0.17	**−0.28**	−0.36, −0.19	**–**	–

ECHILD cohort of isolated cleft lip and/or palate born in NHS England hospitals between 2003 and 2013

^(^a^)^Confidence intervals (CI) were calculated using 1000 bootstraps and account for clustering by home address local authority.

Ass: association; Cond: conditional; ATE: average treatment Effect; ATT: Average Treatment Effect in the treated. Estimates replicated in R and STATA

Of note, conditional RaRs, RiRs, and Δs estimated using multivariable regression models were very similar to the corresponding estimated ATEs (Table 4).

### Sensitivity analyses

#### Restricted follow-up.

When follow-up was restricted to outcomes that occurred up to the end of Year 2, the crude associations were marginally stronger while the causal estimates were very similar to those found using the full follow-up information (Table 5). See S9 Table for a detailed breakdown of the pupils included in this analysis.

**Table 5:** Estimated causal rate ratios (RR) and causal odds ratios (OR) of the effect of Special Education Needs Support versus No provision for unplanned hospitalization and absences restricted to follow-up up to end of Year Two, by estimation methods.

Outcome	Exposure	N	(%)	Causal contrast	Estimation Method					
					Regression		Inverse Probability Weighting		G-computation	
					RR	95% CI	RR	95% CI^(a)^	RR	95% CI^(a)^
**Unplanned Hospital Utilization**	**All**	**6359**	(100.0)	Crude Ass.	1.43	1.05, 1.94				
	No provision	4350	(68.4)	Cond. Ass.	1.05	0.89, 1.24				
	Special education Needs Support	2009	(31.5)	ATE			1.32	1.03, 1.68	1.13	0.83, 1.62
				ATT			1.26	0.89, 1.76	1.06	0.70, 1.62
**Medical Absences**	**All**	**6354**	(100.0)	Crude Ass.	1.42	1.35, 1.49				–
	No provision	4347	(68.4)	Cond. Ass.	1.07	1.20, 1.08				–
	Special education Needs Support	2007	(31.6)	ATE			1.13	1.05, 1.21	1.14	1.07, 1.20
				ATT			1.09	1.00, 1.19	1.11	1.03, 1.20
**Unauthorised Absences**	**All**	**6354**	(100.0)	Crude Ass.	1.31	1.16, 1.47				–
	No provision	4347	(68.4)	Cond. Ass.	1.07	0.93, 1.22				–
	Special education Needs Support	2007	(31.6)	ATE			0.91	0.77, 1.07	0.96	0.84, 1.12
				ATT			0.92	0.71, 1.19	0.99	0.84, 1.12
					OR	95% CI	OR	95% CI^**(b)**^	OR	95% CI^**(b)**^
**Persistent Absences**	**All**	**6354**	(100.0)	Crude Ass.	2.11	1.81, 2.47				–
	No provision	4347	(68.4)	Cond. Ass.	1.13	0.93, 1.37				–
	Special education Needs Support	2007	(31.6)	ATE			1.08	0.86, 1.35	1.13	0.96, 1.33
				ATT			1.05	0.79, 1.39	1.07	0.92, 1.33

ECHILD cohort of isolated cleft lip and/or palate born in NHS England hospitals between 2003 and 2013.

^(^a^)^Confidence intervals (CI) calculated using 1000 bootstraps and accounted for clustering by home address local authority. Ass: association; Cond: conditional; ATE: average treatment effect; ATT: Average Treatment Effect in the treated

#### Complete records analysis.

Children with complete confounder information were more likely to be from later academic years than those without, but they were generally very similar with respect to the other characteristics (S10 Table and S11 Table). This is reflected in the updated ATE and ATT estimates which are very similar to the original ones (S12 and S13 Table).

#### Disaggregation of unplanned hospital utilisation.

We disaggregated unplanned hospital utilisation into accident and emergency visits and admissions to reflect differential care pathways because admissions to APC require clinical input whilst visits to AE do not. When focusing on AE visits, the ATE and ATT estimate are similar to the original analysis. When focussing on unplanned hospital utilisation to APC admissions, we find elevated rates for children assigned to SEN compared to No Support, RaR_ATE_g _= 1.50 (1.27, 1.78) and RaR_ATE_g _= 1.31(1.09, 1.55) and RaR ATT_ipw_ = 1.44 (1.19, 1.74). The corresponding ATT results were similar to their ATE results. See S14 Table for further details.

## Discussion

### Key results

We developed a national cohort of 6,601 children with CLP without additional major congenital anomalies; in this population, 30.4% were recorded to have SEN Support and 3.7% to EHCP in the Year 1. Due to lack of positivity in comparisons involving EHCP, we limited our analyses to the comparison of No Support versus SEN Support when analysing health and educational outcomes. Observed rates of unplanned hospital utilisation and absences during follow-up were higher amongst children with recorded SEN support compared to None, whilst KS1 and KS2 math scores were lower in the same comparison sets. Using multiple methods to account for confounding-by-indication, we found little evidence of a causal effect of recorded SEN Support on unplanned hospitalisation utilisation or persistent absences when in reference to no recorded support. These multiple methods also found that SEN support would increase medical absences but be protective for unauthorised absences when compared to No Support. Finally, we found that assignment to SEN Support would decreases KS1 and KS2 math z-scores in comparison to No Support, although not to the same extent as it would appear by simple comparison of the observed data.

### Analytical strategy implications

We used the target trial emulation framework to guide our study design and analysis when using administrative data to estimate the causal effects of SEN provision on various childhood health and education outcomes. For a causal interpretation of our results, we assumed to have measured all relevant confounders (“no unmeasured confounding”), in addition to having clearly defined the exposure (“counterfactual consistency”), and to have sufficient data to estimate all potential outcomes without extrapolation outside of the information held in the data (“positivity”). With regards to confounding, we were guided by our assumed DAG to identify confounding biases needing control. The variables identified there were available in the administrative databases, but we cannot discount the fact that they are likely to be affected by measurement/misclassification error. With regards to consistency, recorded SEN provision is well-defined for questions concerning the impact of the *intention* of delivering the provision. It would not be well-defined for questions concerning the *delivery* of SEN without making the additional assumptions that recording actually leads to the planned delivery and that alternative formats of that delivery have the same impact on the outcomes.

Our exposure had three levels, None, SEN Support and EHCP as recoded in Year 1. We found lack of positivity for comparisons involving EHCP given the near-zero probability of being assigned to EHCP for the majority of the cohort. For this reason, we concentrated on comparing recording of SEN Support versus None. We adopted alternative methods to estimate the causal contrasts of interest to be able to compare (triangulate) results in the light of the different parametric assumptions they involve. We found that a simple implementation of IPW, with no interactions or higher order modelling of the confounders, gave slightly different results from those obtained using AIPW with Lasso selection of both PS and outcome model specifications. The results from g-computation seemed to agree more with the latter, and gave more precise estimates, attributable to the stronger parametric assumptions.

### Strengths

Our population consisted of a total of 6,601 pupils with CLP without other major congenital anomalies, who started Year One between the academic years of 2008/2009 and 2018/2019. We externally validated our cohort against published data to understand its representativeness; whilst we found higher rates of CLP at birth, our patterns in severity and mathematics scores were similar which suggest some generalisability in our cohort. Related, our use of the ECHILD dataset allows us to have a relatively representative cohort given that ECHILD is “near universal” in terms of state education and NHS funded hospital activity.

Another strength of our study is our use of the target trial emulation framework to reduce confounding by indication firstly by creating an ideal prospective trial and then map the observational data to this ideal trial allowing the appropriate cohort selection in terms of (in)eligibility, and the appropriate start of follow-up. To ensure comparability between multiple categories of SEN provision, we assess the validity of the positivity assumption by evaluating the overlap of propensity distributions between all pair-wise comparisons of SEN provision categories. This led to concentrating one comparison and avoiding extrapolations outside the information held in the data.

Furthermore, we used a variety of methods to triangulate the estimated impact of SEN provision on unplanned hospitalisations, absences and academic performance. The use of multiple methods (IPW, the parametric g-formula computation and AIPW) allowed us to present alternative estimates and to consider their robustness.

### Limitations

Whilst we used a target trial emulation framework and a variety of complementary methods to triangulate our estimates, unmeasured confounding could still impact our results as with all observational studies. We considered adopting an instrumental variable approach to avoid the assumption of no unmeasured confounding [6]; however, no adequate instrumental variable was identified in the data for this specific population, where our candidate instrumental variables included: local authority variation (where we found minimal differences after adjusting for individual circumstances), month of birth and time-varying SEN policy changes introduced in 2014.

Whilst the ECHILD dataset has a wide range of individual and school characteristics available, its administrative nature means that it did not provide access to a variety of predictors such as family composition, the early childhood caring environment (such as the home learning environment, parenting style, and childcare access) which are shown to explain Key Stage 1 and Key Stage 2 attainment outcomes [28]. A further example of uncontrolled confounding is the “treatment-prevalent-paradox” where treatment may be given but the specific reason for providing the treatment is not recorded, therefore leading to unfair comparisons between the groups. Its consequence is that those in the treatment group may be shown to have worse outcomes compared to those without treatment, even after control for measured predictors of treatment and outcome, although it may be widely accepted that without such treatment, the outcome would be much worse [29]. Furthermore, our data were limited by missingness particularly for deprivation and birth characteristics (even if there was a birth record). We assumed our data to be missing at random (MAR) and adopted the missing indicator method to deal with missing values while also controlling for predictors of missingness. The MAR assumption would not be met in our cohort, if the missing mechanism depended on factors not included among the confounders. Given the broad range of included variables, ranging from socio-demographic to clinical, however, we expect the assumption to be justifiable.

Finally, we would like to acknowledge that a recording of No Provision, SEN Support or EHCP in administrative educational records does not necessarily reflect their precise implementation. For example, there could be failure to deliver provision, or teaching staff might prematurely give provision to children who are in the process of applying for provision, or there could be spillover effects from other children in the classroom.

### Interpretation

Estimating the results using a target trial framework shows evidence of no effect of early SEN Support relatively to None in terms of unplanned hospital utilisation and persistent absences. These results contrast with the apparent increase with SEN Support compared to No Support in the raw underlying data suggesting controlling for bias reduced these rates differentials. In contrast, there was some indicative evidence that rates of medical and unauthorised absences were – at least partly – affected in opposite directions by SEN Support, with rates respectively increased and reduced by such intervention. One possible explanation for this is that children with early SEN Support (compared to No Provision) are having their absences reclassified from unauthorised to medical in response to schools knowing their need for SEN (although EHCP is specifically designated for health-based needs).

Early SEN Support was not found to rebalance KS1 and KS2 Maths scores, either when taken in isolation or relative to attainment scores in the reception year (age 4–5). This could be due to a variety of reasons; firstly, there were not enough variables to account for all SEN allocation leading to residual confounding. An alternative explanation is that the of intentions of support does not always correspond to actual delivery, perhaps even introducing unintended consequences such as a ceiling effect or introducing lower expectations; such hypothesis would warrant investigation. Moreover, we have estimated the impact of early SEN provision on two measurable outcomes available in ECHILD (health and education); however, our interactions with our participant and patient groups indicate that SEN provision may improve other outcomes not measured within the data such as “improved quality of life”.

Given the considerations above, we would like to make a few suggestions towards the improvement of data for the purpose of enhancing future research in this area in general, not just for children affected by CLP. Firstly, we have used recorded CLP as a proxy for the need for SEN provision, particularly related to health; ideally, the NPD should have a measure of (suspected) need for SEN in a similar manner to the Free School Meal eligibility variable, irrespective of whether the child receives this or not. Secondly, we would like to have a measure of whether or how much SEN provision was delivered to the child. Thirdly, we understand that children are provided SEN for a variety of reasons, many beyond what is currently recorded; therefore, we kindly suggest collecting data about the reason(s) SEN was provided (or suspected). We understand current state funded schools are overstretched; subsequently, we propose such data only be collected or derived passively, for example by analysing free text reports.

## Supporting information

S1 Fig Simplified directed acyclic graph denoting the relationship between our variables. Double arrows (located at social determinants and birth characteristics) denote that they impact all items downstream (i.e., to the right). In this diagram, social determinants include Gender, Ethnic Group, English as a second language, Index Deprivation Affecting Children Index (IDACI), Free School Meal eligibility and Academic Year (to capture time varying changes). In this diagram, birth characteristics include maternal age, birth weight and gestational age.
(TIF)

S2 Fig Mean and 95% confidence intervals for standardised Early Years Foundation Stage Profile Math Scores by Cleft Lip and/or Palate Type: Top:ECHILD source; (b) Fitzsimmons et al (2018). Cleft Lip Only n = 1627; Cleft Palate Only n = 2852; Unilateral Cleft Lip and Palate n = 1630; Bilateral Cleft Lip and Palate n = 431. Bottom:Standardised EYFSP Mathematics scores from Fitzsimons KJ, Copley LP, Setakis E, et al Early academic achievement in children with isolated clefts: a population-based study in England Archives of Disease in Childhood 2018;103:356–362.
(TIF)

S3 Fig Comparison of density distribution of propensity scores for No Support receives compared to SEN support using multiple methods. Log-Reg = Logistic Regression, LASSO = least absolute shrinkage and selection operator, CART = Classification and Regression Tree, SVM = Support Vector Machines.
(TIF)

S4 Fig Comparison of density distribution of propensity scores for SEN receives compared to None using multiple methods. Log-Reg = Logistic Regression, LASSO = least absolute shrinkage and selection operator, CART = Classification and Regression Tree, SVM = Support Vector Machines.
(TIF)

S5 Fig Comparison of density distribution of propensity scores for EHCP in those who received SEN provision using multiple methods. Log-Reg = Logistic Regression, LASSO = least absolute shrinkage and selection operator, CART = Classification and Regression Tree, SVM = Support Vector Machines.
(TIF)

S6 Fig Comparison of density distribution of propensity scores for EHCP in those who received SEN provision using multiple methods. Log-Reg = Logistic Regression, LASSO = least absolute shrinkage and selection operator, CART = Classification and Regression Tree, SVM = Support Vector Machines.
(TIF)

S7 Fig Comparison of density distribution of propensity scores for EHCP in those who received No provision using multiple methods. Log-Reg = Logistic Regression, LASSO = least absolute shrinkage and selection operator, CART = Classification and Regression Tree, SVM = Support Vector Machines.
(TIF)

S8 Fig Comparison of density distribution of propensity scores for EHCP in those who received EHCP using multiple methods. Log-Reg = Logistic Regression, LASSO = least absolute shrinkage and selection operator, CART = Classification and Regression Tree, SVM = Support Vector Machines.
(TIF)

S1 Table Inclusion and exclusion criteria used to define isolated cleft lip and/or palate. Children with cleft lip and/or palate (including bilateral and unilateral): Q35x Q36x Q371, Q373, Q375, Q379, Q370, Q372, Q374, Q378. Exclusion criteria are major congenital anomalies.
(DOCX)

S2 Table List of variables recording special educational needs in the National Pupil Database. (DOCX)

S3 Table Determining unplanned admissions in hospital episode statistics admitted patient care. (DOCX)

S4 Table Determining medical related absences in the National Pupil database. (DOCX)

S5 Table Trial emulation to estimate the causal effect of Special Educational Needs provision by Year 1 on unplanned hospitalisations by Year 6 in children with cleft lip and/or palate (without other congenital anomalies). (DOCX)

S6 Table External comparison of Cleft Lip and Palate rates in this study versus external sources. (DOCX)

S7 Table External comparison of Cleft Lip and Palate types versus external sources. (DOCX)

S8 Table Distribution of predicted propensity scores by propensity score models. (DOCX)

S9 Table Description of children with Key Stage 2 data by recorded Special Education Needs provision. Pupils with Education and Healthcare Plan in Year 1 are not included as they were not analysed due to lack of propensity overlap.
(DOCX)

S10 Table Description of confounders among children with complete confounder data. (DOCX)

S11 Table Description of confounders and outcomes among children with complete confounder data. (DOCX)

S12 Table Estimated causal rate ratios (RaR) and causal risk ratios (RiR) of the effect of Special Educational Needs Support versus No SEN provision for unplanned hospitalization and absences restricted to children with complete confounder data, by estimation methods. (DOCX)

S13 Table Estimated causal mean differences (Δ) of the effect of SEN Support versus No SEN provision for key stage test results by estimation method restricted to follow-up up to end of Year Two. (DOCX)

S14 Table Estimated causal rate ratios (RaR) of the effect of Special Educational Needs Support versus no special educational needs provision for alternative definition of unplanned hospitalization by estimation method. (DOCX)
