# Supplementary figures and images for: The impact of early special educational needs provision on later hospital admissions, school absence and education attainment: A target trial emulation study of children with isolated cleft lip and/or palate
Source: PLoS One. 2025 Jul 16;20(7):e0327720. doi: 10.1371/journal.pone.0327720 (PMC12266429; doi:10.1371/journal.pone.0327720)

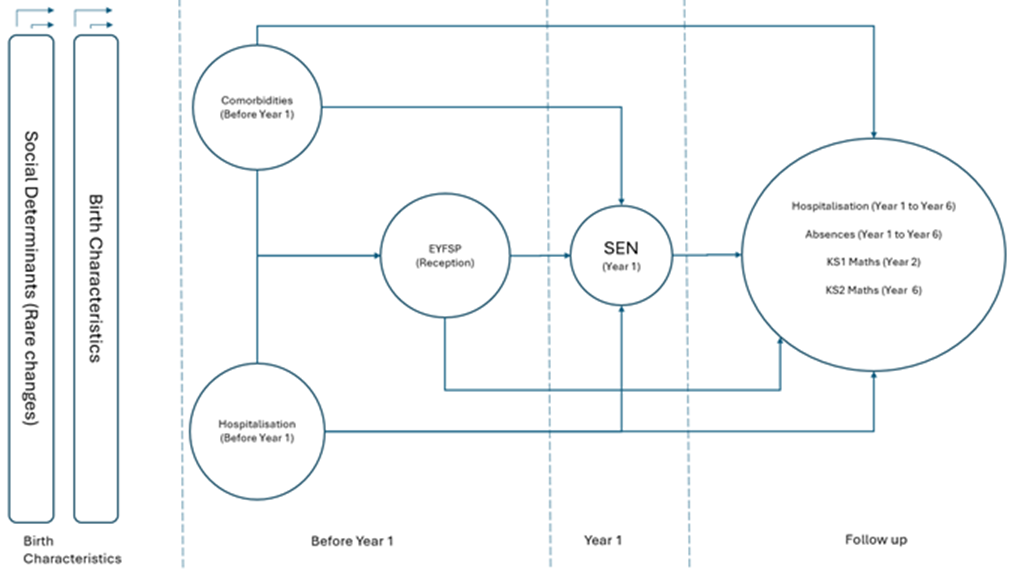

Supplement: S1 Fig — Double arrows (located at social determinants and birth characteristics) denote that they impact all items downstream (i.e., to the right). In this diagram, social determinants include Gender, Ethnic Group, English as a second language, Index Deprivation Affecting Children Index (IDACI), Free School Meal eligibility and Academic Year (to capture time varying changes). In this diagram, birth characteristics include maternal age, birth weight and gestational age. (TIF) [file pone.0327720.s001.tif]

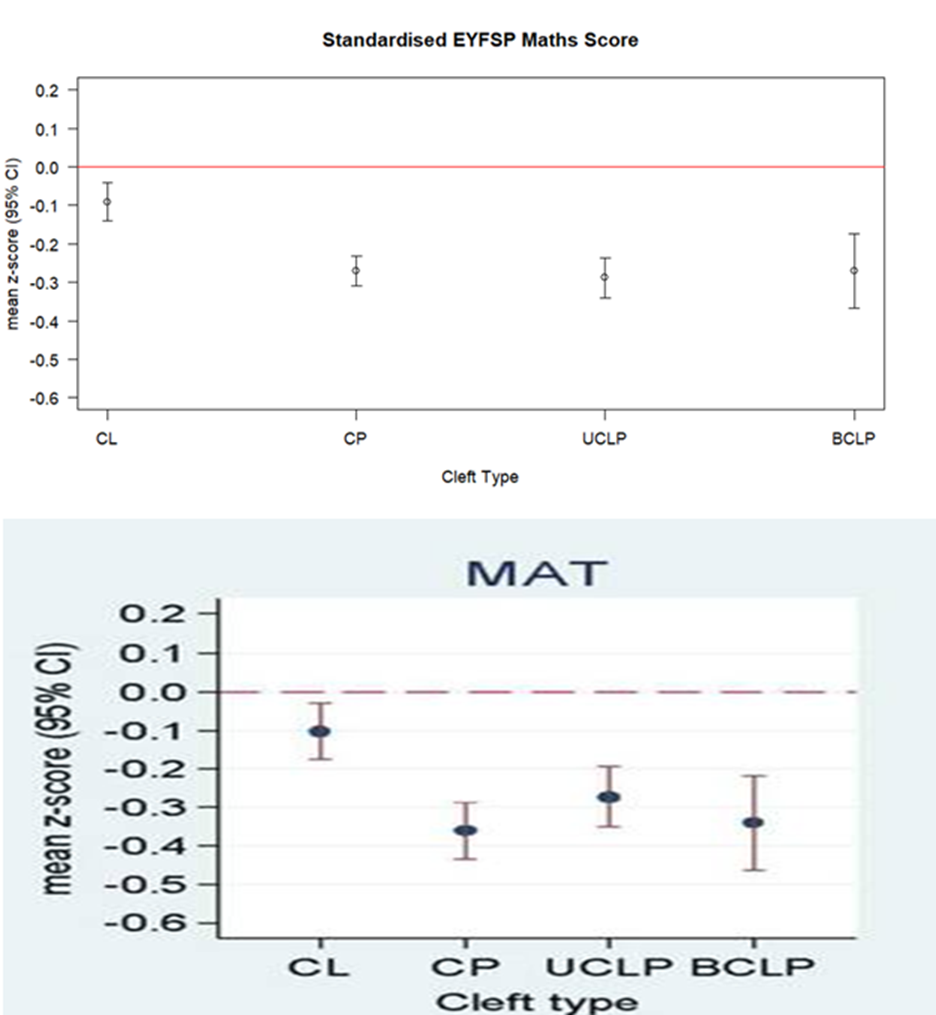

Supplement: S2 Fig — Cleft Lip Only n = 1627; Cleft Palate Only n = 2852; Unilateral Cleft Lip and Palate n = 1630; Bilateral Cleft Lip and Palate n = 431. Bottom:Standardised EYFSP Mathematics scores from Fitzsimons KJ, Copley LP, Setakis E, et al Early academic achievement in children with isolated clefts: a population-based study in England Archives of Disease in Childhood 2018;103:356–362. (TIF) [file pone.0327720.s002.tif]

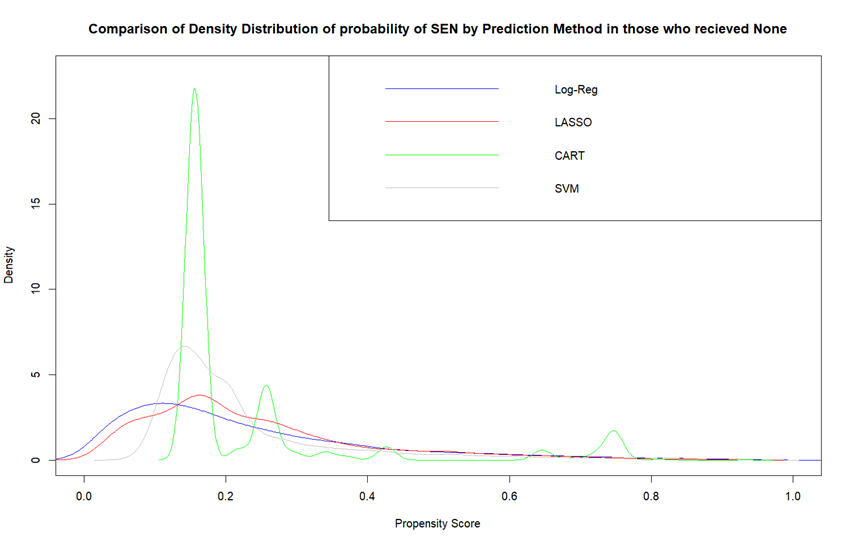

Supplement: S3 Fig — Log-Reg = Logistic Regression, LASSO = least absolute shrinkage and selection operator, CART = Classification and Regression Tree, SVM = Support Vector Machines. (TIF) [file pone.0327720.s003.tif]

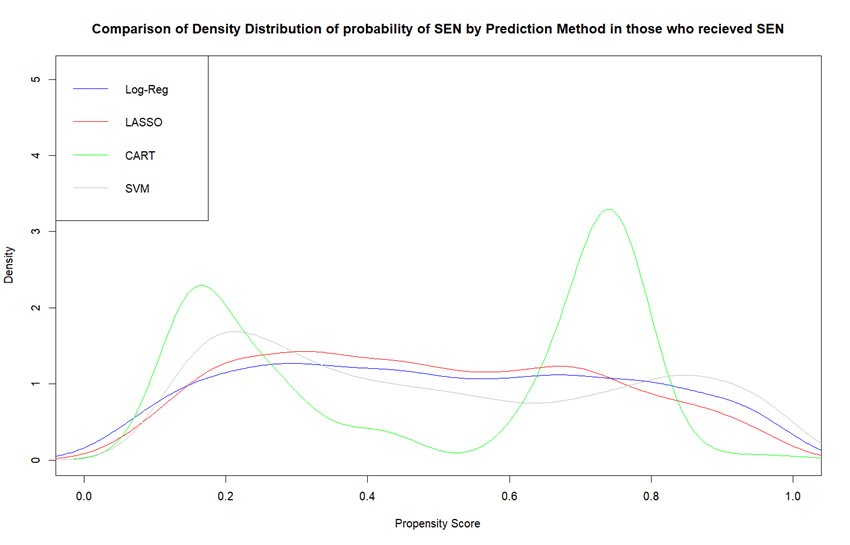

Supplement: S4 Fig — Log-Reg = Logistic Regression, LASSO = least absolute shrinkage and selection operator, CART = Classification and Regression Tree, SVM = Support Vector Machines. (TIF) [file pone.0327720.s004.tif]

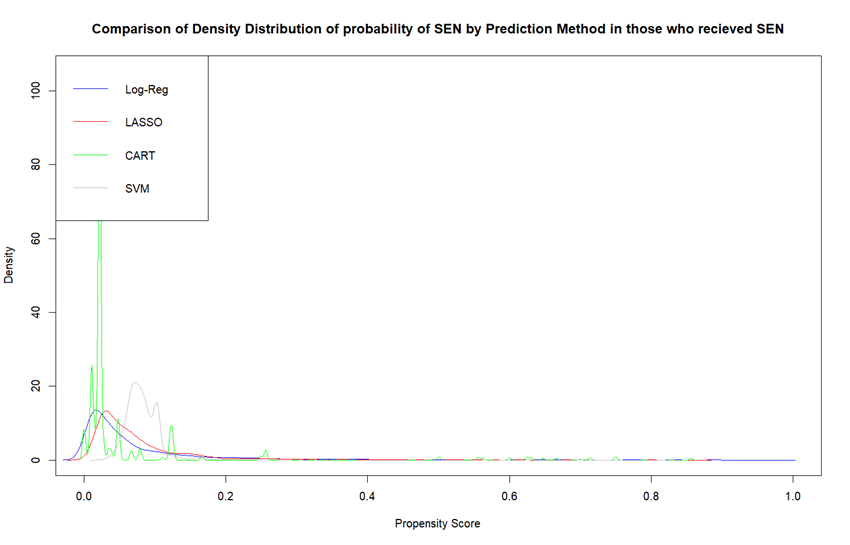

Supplement: S5 Fig — Log-Reg = Logistic Regression, LASSO = least absolute shrinkage and selection operator, CART = Classification and Regression Tree, SVM = Support Vector Machines. (TIF) [file pone.0327720.s005.tif]

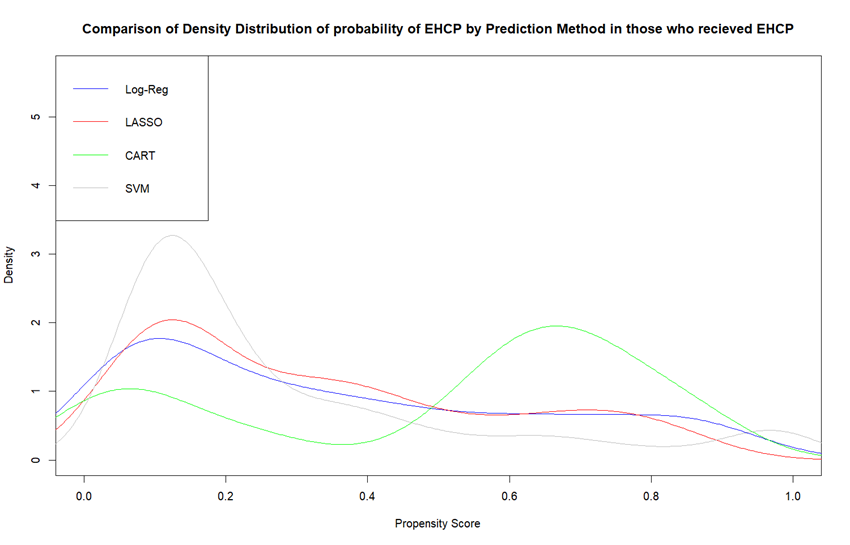

Supplement: S6 Fig — Log-Reg = Logistic Regression, LASSO = least absolute shrinkage and selection operator, CART = Classification and Regression Tree, SVM = Support Vector Machines. (TIF) [file pone.0327720.s006.tif]

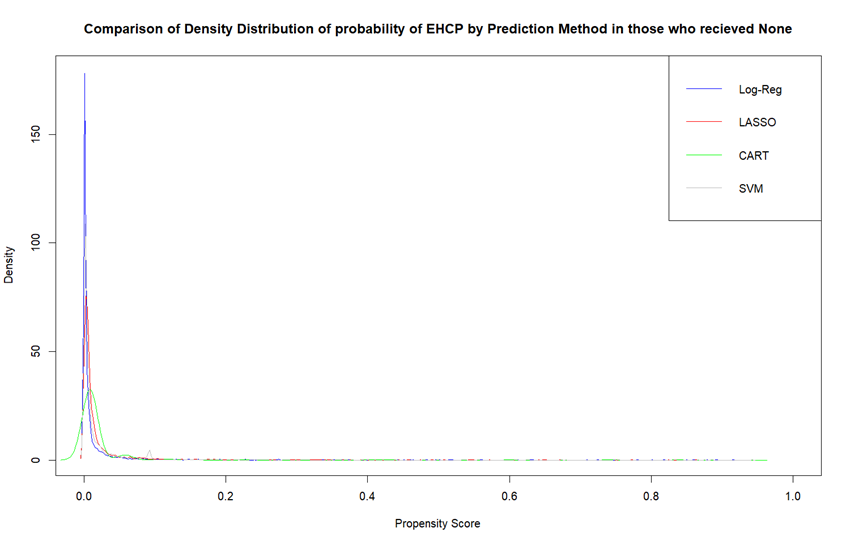

Supplement: S7 Fig — Log-Reg = Logistic Regression, LASSO = least absolute shrinkage and selection operator, CART = Classification and Regression Tree, SVM = Support Vector Machines. (TIF) [file pone.0327720.s007.tif]

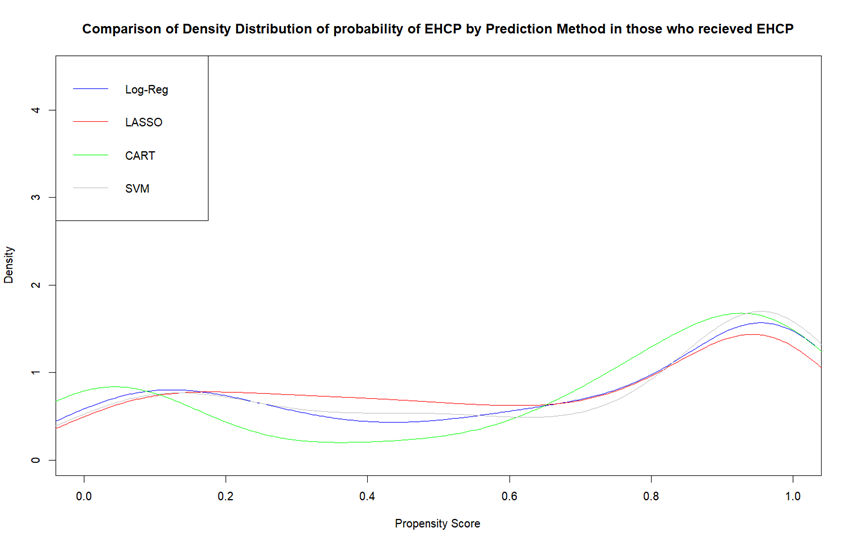

Supplement: S8 Fig — Log-Reg = Logistic Regression, LASSO = least absolute shrinkage and selection operator, CART = Classification and Regression Tree, SVM = Support Vector Machines. (TIF) [file pone.0327720.s008.tif]
