# Supplementary material for: The impact of early special educational needs provision on later hospital admissions, school absence and education attainment: A target trial emulation study of children with isolated cleft lip and/or palate
Source: PLoS One. 2025 Jul 16;20(7):e0327720. doi: 10.1371/journal.pone.0327720 (PMC12266429; doi:10.1371/journal.pone.0327720)
Supplement: S1 Table — Children with cleft lip and/or palate (including bilateral and unilateral): Q35x Q36x Q371, Q373, Q375, Q379, Q370, Q372, Q374, Q378. Exclusion criteria are major congenital anomalies. (DOCX) [file pone.0327720.s009.docx]

| **Condition, by severity** | **ICD-10 codes*** | |
| --- | --- | --- |
|  | **Inclusion** | **Exclusion**** |
| Cleft palate | Q35x | Q-chapter, D215, D821, D1810**, P350, P351, P371  Apart from: Q0461**, Q0782**, Q101, Q102, Q103, Q105, Q135, Q170, Q171, Q172, Q173, Q179, Q174, Q180, Q181, Q182, Q184, Q185, Q186, Q187, Q1880**, Q189, Q2111, Q250 if gestational age <37 weeks, Q2541, Q256 if gestational age <37 weeks, Q261, Q270, Q314, Q320, Q331, Q381, Q382, Q3850**, Q400, Q4021**, Q430, Q4320**, Q4381**, Q4382**, Q523, Q525, Q527, Q53, Q5520**, Q5521**, Q610, Q627, Q633, Q653-Q656, Q662-Q669, Q670-Q678, Q680, Q6821**, Q683-Q685, Q6810**, Q7400**, Q752, Q753, Q760, Q7643, Q765, Q7660**, Q7662**, Q7671**, Q825, Q8280**, Q833, Q845, Q899 |
| Cleft lip | Q36x |  |
| Unilateral cleft lip and palate | Q371, Q373, Q375, Q379 |  |
| Bilateral cleft lip and palate | Q370, Q372, Q374, Q378 |  |

*Identified as a primary or secondary diagnoses in any hospital admission record prior to the start of Year One of school (age 5 at entry); **all cleft lip and/or palate groups also have congenital anomalies (excluding those relating to cleft lip and/or palate) excluded; ICD-10 = International Classification of Diseases version 10
