# Supplementary material for: The impact of early special educational needs provision on later hospital admissions, school absence and education attainment: A target trial emulation study of children with isolated cleft lip and/or palate
Source: PLoS One. 2025 Jul 16;20(7):e0327720. doi: 10.1371/journal.pone.0327720 (PMC12266429; doi:10.1371/journal.pone.0327720)
Supplement: S2 Table — (DOCX) [file pone.0327720.s010.docx]

| **Variable Name in the national pupil database** | **Variable Description as per the national pupil database data dictionary** |
| --- | --- |
| PrimarySENtype | Nature of pupil's primary special educational need. For pupils with a SEN status of E or K their main or primary need and, if appropriate, their secondary need, should be recorded. |
| SecondarySENtype | Nature of pupil's secondary special educational need. For pupils with a SEN status of E or K their main or primary need and, if appropriate, their secondary need, should be recorded. |
| SENProvision | Provision types under the SEN Code of Practice |
| CensusSEN | Provision types under the SEN Code of Practice. |
| SEN_provision | Special Educational Needs provision |
| SENA  SENELK  SENELSE  SENF  SENPS  SEN_ALL  SENAPK  SENSE | - SENA: Does a pupil have SEN - school action? - SENELK: Does pupil have SEN support? - SENELSE: Does pupil have SEN with statement or EHC plan? - SENF: Pupil SEN status - SENPS: Does pupil have SEN - Action Plus or Statemented? - SEN_ALL: Does pupil have SEN with or without statement or EHC plan? - SENAPK: Does pupil have SEN without statement or EHC plan? - SENSE: Does pupil have SEN with statement or EHC plan? |
| LatestSEN | Provision types under the SEN Code of Practice. |
| SENProvisionMajor | Pupil's major SEN provision group based on SEN provision code. |
| SENstatus | Provision types under the SEN Code of Practice. |
| SENUnitIndicator | Indicates if a pupil with SEN in a mainstream school is a member of a SEN Unit (sometimes called special class) |
| SpecialProvisionIndicator | Indicates if a pupil with SEN in a mainstream school is a member of an SEN Unit, special class or resourced provision. |

NPD = national pupil database, SEN = special educational needs
