# Supplementary material for: The impact of early special educational needs provision on later hospital admissions, school absence and education attainment: A target trial emulation study of children with isolated cleft lip and/or palate
Source: PLoS One. 2025 Jul 16;20(7):e0327720. doi: 10.1371/journal.pone.0327720 (PMC12266429; doi:10.1371/journal.pone.0327720)
Supplement: S3 Table — (DOCX) [file pone.0327720.s011.docx]

| **Value of *admimeth* variable in Hospital Episode Statisstics** | **Meaning** | **Action** |
| --- | --- | --- |
| **11, 12, 13** | Planned admission | Planned |
| **21, 22, 23, 24, 25, 28, 2A, 2B, 2D** | Unplanned admission | Unplanned |
| **2C** | Unplanned admission for a baby born at home as intended (available from 2013/14) | Other (birth) |
| **31, 32** | Maternity admission | Other (maternity) |
| **82, 83** | Birth of a baby | Other (birth) |
| **81** | Transfer of any admitted patient from other Hospital Provider other than in an Unplanned | Planned |
| **84** | Admission by Admissions Panel of a High-Security Psychiatric Hospital, patient not entered on the HSPH Admissions Waiting List (available between 1999 and 2006) | Planned |
| **89** | HSPH Admissions Waiting List of a High-Security Psychiatric Hospital (available between 1999 and 2006) | Planned |
| **98** | Not applicable (available from 1996/97) | Other |
| **99** | Not known: a validation error | Other |
