# Supplementary material for: The impact of early special educational needs provision on later hospital admissions, school absence and education attainment: A target trial emulation study of children with isolated cleft lip and/or palate
Source: PLoS One. 2025 Jul 16;20(7):e0327720. doi: 10.1371/journal.pone.0327720 (PMC12266429; doi:10.1371/journal.pone.0327720)
Supplement: S4 Table — (DOCX) [file pone.0327720.s012.docx]

| **Absence Reason in the National Pupil Database** | **Meaning** | **Action** |
| --- | --- | --- |
| C | Number of authorised sessions missed during the academic year as pupil is absent due to other authorised circumstances. | Exclude |
| E | Number of authorised sessions missed during the academic year as pupil is excluded, with no alternative provision made. | Exclude |
| F | Number of authorised sessions missed during the academic year due to agreed extended family holiday. | Exclude |
| G | Number of unauthorised sessions missed during the academic year as pupil is on a family holiday, not agreed, or is taking days in excess of an agreed family holiday. | Include |
| H | Number of authorised sessions missed during the academic year due to agreed family holiday. | Exclude |
| I | Number of authorised sessions missed during the academic year due to Illness (NOT medical or dental etc. appointments). | Include |
| M | Number of authorised sessions missed during the academic year due to medical/ dental appointments. | Include |
| N | Number of unauthorised sessions missed during the academic year as pupil missed sessions for a reason that has not yet been provided. | Include |
| O | Number of sessions missed during the academic year for an unauthorised absence not covered by any other code/description. | Include |
| R | Number of authorised sessions missed during the academic year due to religious observance. | Exclude |
| S | Number of authorised sessions missed during the academic year due to study leave. | Exclude |
| T | Number of authorised sessions missed during the academic year due to traveler absence. | Exclude |
| U | Number of unauthorised sessions missed during the academic year as pupil arrived after registers closed. | Exclude |
