# Supplementary material for: The impact of early special educational needs provision on later hospital admissions, school absence and education attainment: A target trial emulation study of children with isolated cleft lip and/or palate
Source: PLoS One. 2025 Jul 16;20(7):e0327720. doi: 10.1371/journal.pone.0327720 (PMC12266429; doi:10.1371/journal.pone.0327720)
Supplement: S5 Table — (DOCX) [file pone.0327720.s013.docx]

|  | **Ideal target trial** | **Emulated target trial** |
| --- | --- | --- |
| **Eligibility criteria** | Geography: England  Started Year 1 between 2008/2009 and 2018/2019.  Diagnosed with cleft lip and/or palate prior to Year One, and not affected by other major anomalies  Born in England | Geography: England  Started Year 1 in a state school between 2008/2009 and 2018/19.  Identified in hospital episode statistics with cleft lip and/or palate before start of Year 1, and not recorded with other major anomalies  Has a birth record in Hospital Episode Statistics Linked to the National Pupil database |
| **Recruitment period** | Started Year One in between the academic years 2008/2009 and 2018/2019 | Started Year One between the academic years 2008/2009 and 2018/2019 |
| **Follow-up duration** | From: Randomization to the intervention  To: the end of primary school OR loss of follow-up (e.g., emigration) OR death OR end of study | From: January Census in Year One  To: the end of primary school OR loss of follow-up in the national pupil database OR death OR  end of study/end of data (31 August 2019) |
| **Outcome(s)** | Unplanned hospital utilisation as defined by days in admitted patient care or accident and emergency  Medical related absences as defined using half-day sessions  Unauthorised absences as defined using half-day sessions  Persistent absences defined as >10% of potential half-day sessions  Key Stage 1 and Key Stage 2 standardized scores in Mathematics | Unplanned hospital utilisation as defined by days in admitted patient care or accident and emergency Medical related absences as defined using half-day sessions  Unauthorised absences as defined using half day sessions  Persistent absences defined as >10% of potential half-day sessions  Key Stage 1 and Key Stage 2 standardized scores in Mathematics |
| **Interventions to be compared** | One of three categories of Special Educational Needs (SEN) (none, SEN Support, Education and Healthcare Plan) to be delivered following randomization (between start and end of Year 1) | One of three categories of Special Educational Needs (SEN) (none, SEN Support, Education and Healthcare Plan) as recorded by the January census in Year 1 |
| **Causal contrasts** | The average treatment effect (ATE) and average treatment effect in the treated (ATT) of initiating SEN versus non-initiating SEN (and of initiating Education and Healthcare Plan versus initiating SEN Support and initiating an Education and Healthcare Plan versus None) by Year 1 on the number of:   1. unplanned hospital days 2. medical absences 3. unauthorised absences   expressed as rate ratios.  As above for:   1. persistent absences   expressed as a risk ratio.  As above for:   1. Key Stage 1 standardized Math scores 2. Key Stage 2 standardized Math scores 3. Key Stage 1 standardized progress from pre-school test (EYFSP) in Math scores 4. Key Stage 2 standardized progress from pre-school test (EYFSP) in Mats scores   expressed as a mean difference. | Same as for the target trial |
| **Analysis plan** | Poisson or Negative Binomial Regression (depending on the degree of overdispersion) of the number of events accountings for duration of follow-up for outcomes 1-3  Logistic regression for outcome 4  Linear regression for outcomes 5-8  Clustering by school and/or local authority to be dealt with robust inference. | Appropriate methods for confounding adjustment (regression adjustment, g-computation, IPW and AIPW) for the same outcome models as for the target trial.    Clustering by school and/or local authority to be dealt with using robust inference. |
