# Supplementary material for: The impact of early special educational needs provision on later hospital admissions, school absence and education attainment: A target trial emulation study of children with isolated cleft lip and/or palate
Source: PLoS One. 2025 Jul 16;20(7):e0327720. doi: 10.1371/journal.pone.0327720 (PMC12266429; doi:10.1371/journal.pone.0327720)
Supplement: S6 Table — (DOCX) [file pone.0327720.s014.docx]

| **Year** | **Births** | **Cleft Lip and Cases** | **ECHILD Point Estimate** | **Cleft Registry and Audit Network (15 in 10,000)** |
| --- | --- | --- | --- | --- |
| 1997 | 433773 | 790 | 0.001817918 | Not Available |
| 1998 | 575416 | 1073 | 0.001861267 | Not Available |
| 1999 | 568168 | 1056 | 0.001855157 | Not Available |
| 2000 | 551622 | 971 | 0.00175717 | Not Available |
| 2001 | 556083 | 1019 | 0.001829108 | Not Available |
| 2002 | 569455 | 1034 | 0.00181248 | Not Available |
| 2003 | 576680 | 1108 | 0.001917658 | Not Available |
| 2004 | 594058 | 1138 | 0.001911975 | Not Available |
| 2005 | 600766 | 1152 | 0.001913882 | Not Available |
| 2006 | 617080 | 1239 | 0.00200382 | Not Available |
| 2007 | 631897 | 1236 | 0.001952196 | Not Available |
| 2008 | 658128 | 1319 | 0.002000161 | Not Available |
| 2009 | 655710 | 1305 | 0.001986256 | 0.00151 |
| 2010 | 673814 | 1346 | 0.001993602 | 0.00151 |
| 2011 | 675571 | 1380 | 0.002038552 | 0.00151 |
| 2012 | 678177 | 1412 | 0.002077726 | 0.00151 |
| 2013 | 651284 | 1320 | 0.002022666 | 0.00151 |
| 2014 | 639756 | 1244 | 0.001940718 | 0.00151 |
| 2015 | 643319 | 1278 | 0.001982634 | 0.00151 |
| 2016 | 643516 | 1218 | 0.001889151 | 0.00151 |
| 2017 | 632055 | 1187 | 0.001874481 | 0.00151 |
| 2018 | 609593 | 1095 | 0.00179306 | 0.00151 |
| 2019 | 592061 | 975 | 0.001644082 | 0.00151 |
