# Supplementary material for: The impact of early special educational needs provision on later hospital admissions, school absence and education attainment: A target trial emulation study of children with isolated cleft lip and/or palate
Source: PLoS One. 2025 Jul 16;20(7):e0327720. doi: 10.1371/journal.pone.0327720 (PMC12266429; doi:10.1371/journal.pone.0327720)
Supplement: S7 Table — (DOCX) [file pone.0327720.s015.docx]

| **Cleft Type** | **Numbers in this study** | **Estimate in this Study** | **Estimate by Cleft Registry and Audit Network** |
| --- | --- | --- | --- |
| Cleft Lip Only | 5000 | 18% | 25% |
| Cleft Palate Only | 14754 | 54% | 42% |
| Unilateral Cleft Lip and Palate | 5816 | 21% | 21% |
| Bilateral Cleft Lip and Palate | 1804 | 7% | 9% |
| Other | - | - | ~3% |
