# Supplementary material for: The impact of early special educational needs provision on later hospital admissions, school absence and education attainment: A target trial emulation study of children with isolated cleft lip and/or palate
Source: PLoS One. 2025 Jul 16;20(7):e0327720. doi: 10.1371/journal.pone.0327720 (PMC12266429; doi:10.1371/journal.pone.0327720)
Supplement: S8 Table — (DOCX) [file pone.0327720.s016.docx]

|  |  | Propensity for Special Educational Needs Support relative to No Provision | | | | |  |
| --- | --- | --- | --- | --- | --- | --- | --- |
|  |  | < 0.2 | 0.2 to <0.4 | 0.4 to < 0.6 | 0.6 to < 0.8 | 0.8 to 1.0 | Total |
| Special Education Needs Provision Type | No Provision | 2430  (56%) | 1252  (29%) | 429  (10%) | 188  (4%) | 49  (1%) | 4348  (100%) |
|  | Special Educational Needs Provision | 293  (15%) | 494  (25%) | 455  (23%) | 438  (22%) | 329  (16%) | 2009  (100%) |

|  |  | Propensity for Education and Healthcare Plan relative to No Provision | | | | |
| --- | --- | --- | --- | --- | --- | --- |
|  |  | < 0.2 | 0.2 to <0.4 | 0.4 to < 0.6 | 0.6 to 1.0* | Total |
| Special Education Needs Provision Type | No Provision | 4230  (97.3%) | 78  (1.79%) | 22  (0.51%) | 18  (0.4%) | 4348  (100%) |
|  | Education and Healthcare Plan | 53  (22.0%) | 25  (10.3%) | 21  (8.6%) | 143  (59.1%) | 242  (100%) |

|  |  | Propensity for Education and Healthcare Plan relative to Special Education Needs Provision | | | | |
| --- | --- | --- | --- | --- | --- | --- |
|  |  | < 0.2 | 0.2 to <0.4 | 0.4 to < 0.6 | 0.6 to 1.0* | Total |
| Special Education Needs Provision Type | Special Education Needs Provision | 1816  (90%) | 144  (7%) | 27  (1%) | 22  (2%) | 2009  (100%) |
|  | Education and Healthcare Plan | 100  (41%) | 47  (19%) | 37  (15%) | 58  (25%) | 242  (100%) |

*Bin interval is wider to reduce risk of disclosure
