# Supplementary material for: The impact of early special educational needs provision on later hospital admissions, school absence and education attainment: A target trial emulation study of children with isolated cleft lip and/or palate
Source: PLoS One. 2025 Jul 16;20(7):e0327720. doi: 10.1371/journal.pone.0327720 (PMC12266429; doi:10.1371/journal.pone.0327720)
Supplement: S9 Table — Pupils with Education and Healthcare Plan in Year 1 are not included as they were not analysed due to lack of propensity overlap. (DOCX) [file pone.0327720.s017.docx]

|  | | **Special Education Needs Provision Type** | |  |
| --- | --- | --- | --- | --- |
| **Characteristics** | | **No Provision**  (N=2046, 67.3%) | **Special Education Needs Support**  (N=994, 32.6 %) | **Total**  (N=3040, 100%) |
| **Birth characteristics** | |  |  |  |
|  | **Gender** | | |  |
|  | Female | 919 (72.4%) | 351 (27.6%) | 1270 (100.0%) |
|  | Male | 1127 (63.7%) | 643 (36.3%) | 1770 (100.0%) |
|  | **Gestational Age (Weeks)** | | |  |
|  | 34 Weeks or Less | 32 (53.3%) | 28 (46.7%) | 60 (100.0%) |
|  | 35-36 Weeks | 54 (58.7%) | 38 (41.3%) | 92 (100.0%) |
|  | 37-38 Weeks | 270 (63.8%) | 153 (36.2%) | 423 (100.0%) |
|  | 39 Weeks+ | 1031 (71.6%) | 409 (28.4%) | 1440 (100.0%) |
|  | Unknown | 659 (64.3%) | 366 (35.7%) | 1025 (100.0%) |
|  | **Birthweight Category** | | |  |
|  | 2500g-3499g | 834 (68.4%) | 385 (31.6%) | 1219 (100.0%) |
|  | 3500g and higher | 653 (71.3%) | 263 (28.7%) | 916 (100.0%) |
|  | Less than 2500g | 109 (55.1%) | 89 (44.9%) | 198 (100.0%) |
|  | Unknown | 450 (63.6%) | 257 (36.4%) | 707 (100.0%) |
|  | **Maternal Age (Years)** | | |  |
|  | <20 | 136 (56.2%) | 106 (43.8%) | 242 (100.0%) |
|  | 20-24 | 372 (61.9%) | 229 (38.1%) | 601 (100.0%) |
|  | 25-29 | 537 (69.3%) | 238 (30.7%) | 775 (100.0%) |
|  | 30-34 | 559 (71.7%) | 221 (28.3%) | 780 (100.0%) |
|  | 35 or higher | 359 (70.9%) | 147 (29.1%) | 506 (100.0%) |
|  | Unknown | 83 (61.0%) | 53 (39.0%) | 136 (100.0%) |
| **Demographics** | |  |  |  |
|  | **Ethnic Group (latest in the national pupil database)** | | |  |
|  | Recorded as White | 1733 (67.5%) | 833 (32.5%) | 2566 (100.0%) |
|  | Not recorded as White | 313 (66.0%) | 161 (34.0%) | 474 (100.0%) |
|  | **Language Group** | | |  |
|  | Recorded as English | 1823 (67.6%) | 872 (32.4%) | 2695 (100.0%) |
|  | Not recorded as English | 223 (64.6%) | 122 (35.4%) | 345 (100.0%) |
|  | **Income Deprivation Affecting Children Index Quintile** | | |  |
|  | (Most Deprived) 1 | 428 (55.7%) | 341 (44.3%) | 769 (100.0%) |
|  | 2 | 441 (66.1%) | 226 (33.9%) | 667 (100.0%) |
|  | 3 | 412 (70.5%) | 172 (29.5%) | 584 (100.0%) |
|  | 4 | 396 (72.4%) | 151 (27.6%) | 547 (100.0%) |
|  | (Least Depried) 5 | 363 (78.1%) | 102 (21.9%) | 465 (100.0%) |
|  | Unknown | 6 (75.0%) | 2 (25.0%) | 8 (100.0%) |
|  | **Free School Meal Eligibility** | | |  |
|  | Not Eligible | 1738 (71.7%) | 685 (28.3%) | 2423 (100.0%) |
|  | Eligible | 308 (49.9%) | 309 (50.1%) | 617 (100.0%) |
|  | **Academic Year** |  |  |  |
|  | 2008/2009 | 297 (66.7%) | 148 (33.3%) | 445 (100.0%) |
|  | 2009/2010 | 319 (65.2%) | 170 (34.8%) | 489 (100.0%) |
|  | 2010/2011 | 336 (67.2%) | 164 (32.8%) | 500 (100.0%) |
|  | 2011/2012 | 330 (64.2%) | 184 (35.8%) | 514 (100.0%) |
|  | 2012/2013 | 368 (69.8%) | 159 (30.2%) | 527 (100.0%) |
|  | 2013/2014 | 396 (70.1%) | 169 (29.9%) | 565 (100.0%) |
| **Clinical** | |  |  |  |
|  | **Type of Cleft** | | |  |
|  | Cleft Lip only | 593 (78.8%) | 160 (21.2%) | 753 (100.0%) |
|  | Cleft Palate only | 860 (65.7%) | 449 (34.3%) | 1309 (100.0%) |
|  | Unilateral Cleft Lip and Plate | 486 (61.6%) | 303 (38.4%) | 789 (100.0%) |
|  | Bilateral Cleft Lip and Palate | 107 (56.6%) | 82 (43.4%) | 189 (100.0%) |
|  | **Chronic Condition - Any** | | |  |
|  | No | 1333 (74.2%) | 463 (25.8%) | 1796 (100.0%) |
|  | Yes | 713 (57.3%) | 531 (42.7%) | 1244 (100.0%) |
|  | **Chronic Condition – Blood Cancer** | | |  |
|  | No | 2020 (67.6%) | 969 (32.4%) | 2989 (100.0%) |
|  | Yes | 26 (51.0%) | 25 (49.0%) | 51 (100.0%) |
|  | **Chronic Condition – Mental Health Behaviour** | | |  |
|  | No | 2029 (67.8%) | 964 (32.2%) | 2993 (100.0%) |
|  | Yes | 17 (36.2%) | 30 (63.8%) | 47 (100.0%) |
|  | **Chronic Condition - Endocrine Digestive Renal Genitourinary** | | |  |
|  | No | 1949 (68.0%) | 918 (32.0%) | 2867 (100.0%) |
|  | Yes | 97 (56.1%) | 76 (43.9%) | 173 (100.0%) |
|  | **Chronic Condition – Non-specific Codes** | | |  |
|  | No | 1997 (69.0%) | 898 (31.0%) | 2895 (100.0%) |
|  | Yes | 49 (33.8%) | 96 (66.2%) | 145 (100.0%) |
| **School-related** | |  |  |  |
|  | **Early Years Foundation Profile - All - (z-score)** | | |  |
|  | Median | 0.2 | -0.7 | 0.0 |
|  | Q1, Q3 | -0.3, 0.7 | -1.4, 0.0 | -0.7, 0.5 |
|  | **Relative Age** | | |  |
|  | 5 years 11 months old | 178 (69.3%) | 79 (30.7%) | 257 (100.0%) |
|  | 5 years 10 months old | 166 (72.2%) | 64 (27.8%) | 230 (100.0%) |
|  | 5 years 9 months old | 163 (73.8%) | 58 (26.2%) | 221 (100.0%) |
| - | 5 years 8 months old | 164 (66.1%) | 84 (33.9%) | 248 (100.0%) |
|  | 5 years 7 months old | 190 (70.4%) | 80 (29.6%) | 270 (100.0%) |
|  | 5 years 6 months old | 154 (74.8%) | 52 (25.2%) | 206 (100.0%) |
|  | 5 years 5 months old | 153 (68.0%) | 72 (32.0%) | 225 (100.0%) |
|  | 5 years 4 months old | 169 (66.5%) | 85 (33.5%) | 254 (100.0%) |
|  | 5 years 3 months old | 202 (68.0%) | 95 (32.0%) | 297 (100.0%) |
|  | 5 years 2 months old | 150 (60.2%) | 99 (39.8%) | 249 (100.0%) |
|  | 5 years 1 months old | 184 (62.6%) | 110 (37.4%) | 294 (100.0%) |
|  | 5 years 0 months old | 173 (59.9%) | 116 (40.1%) | 289 (100.0%) |
