# Supplementary material for: The impact of early special educational needs provision on later hospital admissions, school absence and education attainment: A target trial emulation study of children with isolated cleft lip and/or palate
Source: PLoS One. 2025 Jul 16;20(7):e0327720. doi: 10.1371/journal.pone.0327720 (PMC12266429; doi:10.1371/journal.pone.0327720)
Supplement: S10 Table — (DOCX) [file pone.0327720.s018.docx]

| **Characteristics** | | **No Provision**  (N=3312, 67.5%) | **Special Educational Needs Support**  (N=1433, 29.2%) | **Total**  (N=4909, 100%) |
| --- | --- | --- | --- | --- |
| **Birth characteristics** | | | | |
|  | **Gender** | | |  |
|  | Female | 1498 (73.1%) | 505 (24.6%) | 2003 (100.0%) |
|  | Male | 1814 (63.4%) | 928 (32.4%) | 2742 (100.0%) |
|  | **Gestational Age (Weeks)** | | |  |
|  | 34 Weeks or Less | 86 (50.3%) | 69 (40.4%) | 155 (100.0%) |
|  | 35-36 Weeks | 146 (62.7%) | 77 (33.0%) | 223 (100.0%) |
|  | 37-38 Weeks | 627 (63.3%) | 333 (33.6%) | 960 (100.0%) |
|  | 39 Weeks+ | 2453 (69.8%) | 954 (27.1%) | 3407 (100.0%) |
|  | **Birthweight Category** | | |  |
|  | 2500g-3499g | 1728 (65.9%) | 799 (30.5%) | 2527 (100.0%) |
|  | 3500g and higher | 1353 (73.0%) | 464 (25.0%) | 1817 (100.0%) |
|  | Less than 2500g | 231 (53.5%) | 170 (39.4%) | 401 (100.0%) |
|  | **Maternal Age (Years)** | | |  |
|  | <20 | 210 (61.2%) | 133 (38.8%) | 343 (100.0%) |
|  | 20-24 | 662 (65.2%) | 353 (34.8%) | 1015 (100.0%) |
|  | 25-29 | 890 (69.7%) | 386 (30.3%) | 1276 (100.0%) |
|  | 30-34 | 931 (73.7%) | 332 (26.3%) | 1263 (100.0%) |
|  | 35 or Higher | 619 (73.0%) | 229 (27.0%) | 848 (100.0%) |
| **Demographics** | |  |  |  |
|  | **Ethnic Group (latest in the national pupil database)** | | |  |
|  | Recorded as White | 2755 (67.7%) | 1191 (29.2%) | 3946 (100.0%) |
|  | Not recorded as White | 557 (66.5%) | 242 (28.9%) | 799 (100.0%) |
|  | **Language Group** |  |  |  |
|  | Recorded as English | 2864 (67.4%) | 1249 (29.4%) | 4113 (100.0%) |
|  | Not recorded as English | 448 (68.0%) | 184 (27.9%) | 632 (100.0%) |
|  | **Income Deprivation Affecting Children Index Quintile** | | |  |
|  | (Most Deprived) 1 | 776 (59.3%) | 498 (38.1%) | 1274 (100.0%) |
|  | 2 | 700 (65.0%) | 340 (31.6%) | 1040 (100.0%) |
|  | 3 | 631 (68.7%) | 254 (27.7%) | 885 (100.0%) |
|  | 4 | 630 (73.9%) | 192 (22.5%) | 822 (100.0%) |
|  | (Least Deprived) 5 | 575 (76.3%) | 149 (19.8%) | 724 (100.0%) |
|  | **Free School Meal Eligibility** | | |  |
|  | Not Eligible | 2827 (71.1%) | 1018 (25.6%) | 3845 (100.0%) |
|  | Eligible | 485 (51.9%) | 415 (44.4%) | 900 (100.0%) |
|  | **Academic Year** | | |  |
|  | 2008/2009 | 196 (61.2%) | 112 (35.0%) | 308 (100.0%) |
|  | 2009/2010 | 223 (62.3%) | 128 (35.8%) | 351 (100.0%) |
|  | 2010/2011 | 231 (65.6%) | 111 (31.5%) | 342 (100.0%) |
|  | 2011/2012 | 212 (63.3%) | 112 (33.4%) | 324 (100.0%) |
|  | 2012/2013 | 226 (66.3%) | 101 (29.6%) | 327 (100.0%) |
|  | 2013/2014 | 289 (67.2%) | 127 (29.5%) | 416 (100.0%) |
|  | 2014/2015 | 309 (68.5%) | 121 (26.8%) | 430 (100.0%) |
|  | 2015/2016 | 379 (69.3%) | 153 (28.0%) | 532 (100.0%) |
|  | 2016/2017 | 409 (69.6%) | 157 (26.7%) | 566 (100.0%) |
|  | 2017/2018 | 416 (70.9%) | 153 (26.1%) | 569 (100.0%) |
|  | 2018/2019 | 422 (70.3%) | 158 (26.3%) | 580 (100.0%) |
| **Clinical** | |  |  |  |
|  | **Type of Cleft** |  |  |  |
|  | Cleft Lip only | 976 (79.7%) | 230 (18.8%) | 1206 (100.0%) |
|  | Cleft Palate only | 1403 (66.1%) | 624 (29.4%) | 2027 (100.0%) |
|  | Unilateral CLP | 748 (60.6%) | 449 (36.4%) | 1197 (100.0%) |
|  | Bilateral CLP | 185 (56.4%) | 130 (39.6%) | 315 (100.0%) |
|  | **Chronic Condition - Any** | | |  |
|  | No | 2173 (76.2%) | 644 (22.6%) | 2817 (100.0%) |
|  | Yes | 1139 (55.3%) | 789 (38.3%) | 1928 (100.0%) |
|  | **Chronic Condition – Blood Cancer** | | |  |
|  | No | 3269 (67.9%) | 1398 (29.0%) | 4667 (100.0%) |
|  | Yes | 43 (45.7%) | 35 (37.2%) | 78 (100.0%) |
|  | **Chronic Condition – Mental Health Behaviour** | | |  |
|  | No | 3273 (69.4%) | 1333 (28.3%) | 4606 (100.0%) |
|  | Yes | 39 (20.1%) | 100 (51.5%) | 139 (100.0%) |
|  | **Chronic Condition - Endocrine Digestive Renal Genitourinary** | | |  |
|  | No | 3161 (68.6%) | 1319 (28.6%) | 4480 (100.0%) |
|  | Yes | 151 (50.7%) | 114 (38.3%) | 265 (100.0%) |
|  | **Chronic Condition – Non-specific Codes** | | |  |
|  | No | 3211 (70.0%) | 1278 (27.9%) | 4489 (100.0%) |
|  | Yes | 101 (31.4%) | 155 (48.1%) | 256 (100.0%) |
| **School-related** | |  |  |  |
|  | **Early Years Foundation Profile - All - (z-score)** | | |  |
|  | Median | 0.0 | -0.8 | -0.1 |
|  | Q1, Q3 | -0.3, 0.6 | -1.7, -0.1 | -0.8, 0.3 |
|  | **Relative Age** | | |  |
|  | 5 Years and 0 months | 255 (61.7%) | 139 (33.7%) | 394 (100.0%) |
|  | 5 Years and 1 months | 298 (64.4%) | 152 (32.8%) | 450 (100.0%) |
| - | 5 Years and 2 months | 270 (63.8%) | 139 (32.9%) | 409 (100.0%) |
|  | 5 Years and 3 months | 292 (65.9%) | 136 (30.7%) | 428 (100.0%) |
|  | 5 Years and 4 months | 269 (66.1%) | 127 (31.2%) | 396 (100.0%) |
|  | 5 Years and 5 months | 232 (68.6%) | 94 (27.8%) | 326 (100.0%) |
|  | 5 Years and 6 months | 245 (70.6%) | 93 (26.8%) | 338 (100.0%) |
|  | 5 Years and 7 months | 297 (71.2%) | 105 (25.2%) | 402 (100.0%) |
|  | 5 Years and 8 months | 265 (62.4%) | 140 (32.9%) | 405 (100.0%) |
|  | 5 Years and 9 months | 308 (73.5%) | 104 (24.8%) | 412 (100.0%) |
|  | 5 Years and 10 months | 276 (70.8%) | 98 (25.1%) | 374 (100.0%) |
|  | 5 Years and 11 months | 305 (71.9%) | 106 (25.0%) | 411 (100.0%) |
