# Supplementary material for: The impact of early special educational needs provision on later hospital admissions, school absence and education attainment: A target trial emulation study of children with isolated cleft lip and/or palate
Source: PLoS One. 2025 Jul 16;20(7):e0327720. doi: 10.1371/journal.pone.0327720 (PMC12266429; doi:10.1371/journal.pone.0327720)
Supplement: S11 Table — (DOCX) [file pone.0327720.s019.docx]

|  |  | **Special Education Needs Provision type** | | | |
| --- | --- | --- | --- | --- | --- |
| **Outcome** |  | **No Provision**  (N=3312, 67.5%) | **Special Education Needs Support**  (N=1433, 29.2%) | **Education and Healthcare Plan**  (N=164, 3.3%) | **Total**  (N=4909, 100%) |
| **Unplanned Hospital Utilisation** | | | | | |
|  | **N** | **3312** | **1433** | **164** | **4909** |
|  | Total number of days in hospital | 3984 | 2553 | 385 | 6922 |
|  | Total number of follow up days | 4,413,091 | 2,021,710 | 223,444 | 6,658,245 |
|  | Rate per 100,000 days | 90.27 | 127.28 | 172.30 | 103.96 |
| **Medical Absences** | | | | | |
|  | **N** | **3312** | **1431** | **164** | **4907** |
|  | Total number of sessions | 139,504 | 84,842 | 12,498 | 236,844 |
|  | Total number of possible sessions | 4,503,348 | 2,032,845 | 217,291 | 6,753,484 |
|  | Rate per 1000 sessions | 30.9 | 41.7 | 57.5 | 35.1 |
| **Unauthorized Absences** | | | | | |
|  | **N** | **3312** | **1431** | **164** | **4907** |
|  | Total number of sessions | 40,628 | 25,522 | 2,699 | 68,849 |
|  | Total number of possible sessions | 4,503,348 | 2,032,845 | 217,291 | 6,753,484 |
|  | Rate per 1000 sessions | 9.0 | 12.5 | 12.4 | 10.2 |
| **Persistent Absences**^(a)^ | |  |  |  |  |
|  | **N** | **3312** | **1431** | **164** | **4907** |
|  | Number pupils | 240 | 214 | 40 | 494 |
|  | Percentage | 7.2% | 14.9% | 24.4% | 10.1% |
| **Key Stage 1 Math Score** | |  |  |  |  |
|  | N | **2858** | **1251** | **135** | **4244** |
|  | **Median** | 0.1 | -0.7 | - | 0.1 |
|  | (25^th^, 75^th^ centile) | -0.3, 0.3 | -1.2, 0.1 | - | -0.9, 0.2 |
| **Reception to Key Stage 1 Math Progression** | | | | | |
|  | N | **2858** | **1251** | **135** | **4244** |
|  | **Median** | 0.1 | 0.1 | - | 0.1 |
|  | (25^th^, 75^th^ centile) | -0.5, 0.5 | -0.5, 0.6 | - | -0.5, 0.6 |
| **Key Stage 2 Math Score** | | | | | |
|  | N | **1327** | **601** | **37** | **1965** |
|  | **Median** | **0.3** | **-0.7** | **-** | 0.0 |
|  | (25^th^, 75^th^ centile) | -0.5, 0.8 | -1.6, 0.2 | -,- | -0.9, 0.7 |
| **EYFSP to Key Stage 2 Math Progression** | | | | | |
|  | N | **1327** | **601** | **37** | **1965** |
|  | Median | 0.0 | 0.0 | - | 0.0 |
|  | (25^th^, 75^th^ centile) | -0.6, 0.5 | -0.8, 0.7 | -,- | -0.6, 0.6 |
