# Supplementary material for: The impact of early special educational needs provision on later hospital admissions, school absence and education attainment: A target trial emulation study of children with isolated cleft lip and/or palate
Source: PLoS One. 2025 Jul 16;20(7):e0327720. doi: 10.1371/journal.pone.0327720 (PMC12266429; doi:10.1371/journal.pone.0327720)
Supplement: S12 Table — (DOCX) [file pone.0327720.s020.docx]

| **Outcome** | **Exposure** | **N** | **(%)** | **Causal contrast** | **Estimation Method** | | | | | |
| --- | --- | --- | --- | --- | --- | --- | --- | --- | --- | --- |
|  |  |  |  |  | **Regression** | | **Inverse Probability Weighting** | | **G-computation** | |
|  |  |  |  |  | RaR | 95% CI | RaR | 95% CI ^(a)^ | RaR | 95% CI^(a)^ |
| **Unplanned Hospital Utilization** | **All** | **4745** | **100** | Crude Ass. | 1.39 | 1.14, 1.70 |  |  |  |  |
|  | No provision | 3312 | 69.7 | Cond. Ass. | 1.13 | 1.00, 1.28 |  |  |  |  |
|  | Special Education Needs Support | 1433 | 30.3 | ATE |  |  | 1.35 | 1.12, 1.62 | 1.11 | 0.92, 1.33 |
|  |  |  |  | ATT |  |  | 1.28 | 1.03, 1.59 | 1.04 | 0.77, 1.33 |
|  |  |  |  |  |  |  |  |  |  |  |
| **Medical Absences** | **All** | **4743** | **100** | Crude Ass. | 1.34 | 1.27, 1.42 |  |  |  |  |
|  | No provision | 3312 | 69.8 | Cond. Ass. | 1.13 | 1.06,1.20 |  |  |  |  |
|  | Special Education Needs Support | 1431 | 30.2 | ATE |  |  | 1.14 | 1.05,1.23 | 1.14 | 1.06, 1.22 |
|  |  |  |  | ATT |  |  | 1.11 | 1.03,1.20 | 1.09 | 1.00, 1.22 |
|  |  |  |  |  |  |  |  |  |  |  |
| **Unauthorised Absences** | **All** | **4743** | **100** | Crude Ass. | 1.26 | 1.12, 1.41 |  |  |  |  |
|  | No provision | 3312 | 69.8 | Cond. Ass. | 1.00 | 0.89, 1.13 |  |  |  |  |
|  | Special Education Needs Support | 1431 | 30.2 | ATE |  |  | 0.92 | 0.80, 1.06 | 1.03 | 0.91, 1.19 |
|  |  |  |  | ATT |  |  | 0.97 | 0.81, 1.16 | 1.03 | 0.87, 1.19 |
|  |  |  |  |  |  |  |  |  |  |  |
|  |  |  |  |  | RiR | 95% CI | RiR | 95% CI ^(a)^ | RiR | 95% CI^(a)^ |
| **Persistent Absences** | **All** | **4743** | **100** | Crude Ass. | 2.25 | 1.85, 2.73 |  |  |  |  |
|  | No provision | 3312 | 69.8 | Cond. Ass. | 1.26 | 0.99, 1.60 |  |  |  |  |
|  | Special Education Needs Support | 1431 | 30.2 | ATE |  |  | 1.27 | 0.96, 1.67 | 1.25 | 1.01, 1.51 |
|  |  |  |  | ATT |  |  | 1.37 | 1.01, 1.85 | 1.24 | 0.99, 1.51 |
|  |  |  |  |  |  |  |  |  |  |  |

ECHILD cohort of isolated cleft lip and/or palate born in NHS England hospitals between 2003 and 2013. Estimates replicated in R and Stata. Confidence intervals (CI) were estimated (a) using 1000 bootstraps and account for clustering by home address local authority. RiR: Risk Ratio; Ass: association; Cond: conditional; ATE: average treatment Effect; ATT: Average Treatment Effect in the treated.
