# Supplementary material for: The impact of early special educational needs provision on later hospital admissions, school absence and education attainment: A target trial emulation study of children with isolated cleft lip and/or palate
Source: PLoS One. 2025 Jul 16;20(7):e0327720. doi: 10.1371/journal.pone.0327720 (PMC12266429; doi:10.1371/journal.pone.0327720)
Supplement: S13 Table — (DOCX) [file pone.0327720.s021.docx]

| **Outcome** | **Exposure** | **N** | **(%)** | **Causal contrast** | **Estimation Method** | | | | | |
| --- | --- | --- | --- | --- | --- | --- | --- | --- | --- | --- |
|  |  |  |  |  | **Regression** | | **Inverse Probability Weighting** | | **G-computation** | |
|  |  |  |  |  | **Δ** | 95% CI | **Δ** | 95% CI^(a)^ | **Δ** | 95% CI^(a)^ |
| **Key Stage 1- Math** | **All** | **4109** | **100** | Crude Ass. | -0.85 | -0.92, -0.78 |  |  |  |  |
|  | No provision | 2858 | 69.6 | Cond. Ass. | -0.26 | -0.32, -0.20 |  |  |  |  |
|  | Special Education Needs Support | 1251 | 30.4 | ATE |  |  | -0.18 | -0.28, -0.07 | -0.23 | -0.29, -0.17 |
|  |  |  |  | ATT |  |  | -0.34 | -0.44, -0.23 | -0.32 | -0.38, -0.17 |
|  |  |  |  |  |  |  |  |  |  |  |
| **Key Stage 2- Math** | **All** | **1928** | **100** | Crude Ass. | -0.82 | -0.92, -0.71 |  |  |  | - |
|  | No provision | 1327 | 68.8 | Cond. Ass. | -0.30 | -0.40, -0.21 |  |  |  | - |
|  | Special Education Needs Support | 601 | 31.2 | ATE |  |  | -0.28 | -0.43, -0.13 | -0.29 | -0.39, -0.18 |
|  |  |  |  | ATT |  |  | -0.40 | -0.54, -0.25 | -0.35 | -0.46, -0.18 |
|  |  |  |  |  |  |  |  |  |  |  |
| **Early Years Foundation Profile - Key Stage 1 progress- Math**^(b)^ | **All** | **4109** | **100** | Crude Ass. | 0.04 | -0.01,0.10 |  |  |  | - |
|  | No provision | 2858 | 69.6 | Cond. Ass. | -0.26 | -0.32, -0.20 |  |  |  | - |
|  | Special Education Needs Support | 1251 | 30.4 | ATE |  |  | -0.31 | -0.42, -0.19 | -0.25 | -0.32, -0.18 |
|  |  |  |  | ATT |  |  | -0.28 | -0.38, -0.19 | -0.32 | -0.39, -0.18 |
|  |  |  |  |  |  |  |  |  |  |  |
| **Early Years Foundation Profile - Key Stage 2 progress- Math**^(c)^ | **All** | **1928** | **100** | Crude Ass. | **0.00** | -0.09, 0.09 |  |  |  |  |
|  | No provision | 1327 | 68.8 | Cond. Ass. | **-0.29** | -0.39, -0.19 |  |  |  |  |
|  | Special Education Needs Support | 601 | 31.2 | ATE |  |  | **-0.29** | -0.43, -0.15 | **-0.30** | -0.41, -0.19 |
|  |  |  |  | ATT |  |  | **-0.36** | -0.53, -0.20 | **-0.32** | -0.44, -0.19 |
|  |  |  |  |  |  |  |  |  |  |  |

ECHILD cohort of isolated cleft lip and/or palate born in NHS England hospitals between 2003 and 2013. Estimates replicated in R and Stata. Confidence intervals (CI) were calculated using (a) 1000 bootstraps and account for clustering by home address local authority. (b) Difference between standardized Key Stage 1 score and standardized Early Years Foundation Profile score in mathematics. (c) Difference between standardized Key Stage 2 score and standardized Early Years Foundation Profile score in mathematics. Ass: association; Cond: conditional; ATE: average treatment Effect; ATT: Average Treatment Effect in the treated.
