# Supplementary material for: The impact of early special educational needs provision on later hospital admissions, school absence and education attainment: A target trial emulation study of children with isolated cleft lip and/or palate
Source: PLoS One. 2025 Jul 16;20(7):e0327720. doi: 10.1371/journal.pone.0327720 (PMC12266429; doi:10.1371/journal.pone.0327720)
Supplement: S14 Table — (DOCX) [file pone.0327720.s022.docx]

| **Outcome** | **Exposure** | **N** | **(%)** | **Causal contrast** | **Estimation Method** | | | | | |
| --- | --- | --- | --- | --- | --- | --- | --- | --- | --- | --- |
|  |  |  |  |  | **Regression** | | **Inverse Probability Weighting** | | **G-computation** | |
|  |  |  |  |  | RaR | 95% CI | RaR | 95% CI ^(a)^ | RaR | 95% CI^(a)^ |
| **Unplanned Hospital Utilization** | **All** | **6,357** | (100.0) | Crude Ass. | **1.31** | 1.11, 1.53 | **-** | - | **-** | - |
|  | No provision | 4,348 | (68.4) | Cond. Ass. | **1.07** | 0.95, 1.19 | **-** | - | **-** | - |
|  | Special Education Needs Support | 2,009 | (31.6) | ATE | **-** | - | **1.16** | 1.00, 1.34 | **0.99** | 0.87, 1.12 |
|  |  |  |  | ATT | **-** | - | **1.10** | 0.92, 1.33 | **0.95** | 0.79, 1.10 |
|  |  |  |  |  |  |  |  |  |  |  |
| **A&E Hospital Utilization** | **All** | **6,357** | (100.0) | Crude Ass. | **1.12** | 1.03, 1.21 |  |  |  |  |
|  | No provision | 4,348 | (68.4) | Cond. Ass. | **1.01** | 0.92, 1.11 |  |  |  |  |
|  | Special Education Needs Support | 2,009 | (31.6) | ATE | **-** | - | **1.16** | 0.99, 1.35 | **0.96** | 0.84, 1.07 |
|  |  |  |  | ATT | **-** | **-** | **1.07** | 0.96, 1.19 | **0.86** | 0.71, 1.07 |
|  |  |  |  |  |  |  |  |  |  |  |
| **Unplanned Admitted Patient Care** | **All** | **6,357** | (100.0) | Crude Ass. | **1.87** | 1.58, 2.21 |  |  |  |  |
|  | No provision | 4,348 | (68.4) | Cond. Ass. | **1.47** | 1.29, 1.66 |  |  |  |  |
|  | Special Education Needs Support | 2,009 | (31.6) | ATE |  |  | **1.50** | 1.27, 1.78 | **1.31** | 1.09,1.55 |
|  |  |  |  | ATT |  |  | **1.44** | 1.19, 1.74 | **1.25** | 0.96, 1.55 |
|  |  |  |  |  |  |  |  |  |  |  |

ECHILD cohort of isolated cleft lip and/or palate born in NHS England hospitals between 2003 and 2013. Estimates replicated in R and Stata. Confidence intervals (CI) were estimated (a) using 1000 bootstraps and account for clustering by home address local authority. RaR: Rate Ratio; Ass: association; Cond: conditional; ATE: average treatment Effect; ATT: Average Treatment Effect in the treated.
